# The flexible N-terminal motif of uL11 unique to eukaryotic ribosomes interacts with P-complex and facilitates protein translation

**DOI:** 10.1093/nar/gkac292

**Published:** 2022-05-11

**Authors:** Lei Yang, Ka-Ming Lee, Conny Wing-Heng Yu, Hirotatsu Imai, Andrew Kwok-Ho Choi, David K Banfield, Kosuke Ito, Toshio Uchiumi, Kam-Bo Wong

**Affiliations:** School of Life Sciences, Centre for Protein Science and Crystallography, State Key Laboratory of Agrobiotechnology, The Chinese University of Hong Kong, Shatin, Hong Kong, China; School of Life Sciences, Centre for Protein Science and Crystallography, State Key Laboratory of Agrobiotechnology, The Chinese University of Hong Kong, Shatin, Hong Kong, China; School of Life Sciences, Centre for Protein Science and Crystallography, State Key Laboratory of Agrobiotechnology, The Chinese University of Hong Kong, Shatin, Hong Kong, China; Department of Biology, Faculty of Science, Niigata University, Ikarashi 2-8050, Nishi-ku, Niigata 950-2181, Japan; School of Life Sciences, Centre for Protein Science and Crystallography, State Key Laboratory of Agrobiotechnology, The Chinese University of Hong Kong, Shatin, Hong Kong, China; Division of Life Science, Hong Kong University of Science and Technology, Clear Water Bay, Hong Kong, China; Department of Biology, Faculty of Science, Niigata University, Ikarashi 2-8050, Nishi-ku, Niigata 950-2181, Japan; Department of Biology, Faculty of Science, Niigata University, Ikarashi 2-8050, Nishi-ku, Niigata 950-2181, Japan; The Institute of Science and Technology, Niigata University, Ikarashi 2-8050, Nishi-ku, Niigata 950-2181, Japan; School of Life Sciences, Centre for Protein Science and Crystallography, State Key Laboratory of Agrobiotechnology, The Chinese University of Hong Kong, Shatin, Hong Kong, China

## Abstract

Eukaryotic uL11 contains a conserved MPPKFDP motif at the N-terminus that is not found in archaeal and bacterial homologs. Here, we determined the solution structure of human uL11 by NMR spectroscopy and characterized its backbone dynamics by ^15^N–^1^H relaxation experiments. We showed that these N-terminal residues are unstructured and flexible. Structural comparison with ribosome-bound uL11 suggests that the linker region between the N-terminal domain and C-terminal domain of human uL11 is intrinsically disordered and only becomes structured when bound to the ribosomes. Mutagenesis studies show that the N-terminal conserved MPPKFDP motif is involved in interacting with the P-complex and its extended protuberant domain of uL10 *in vitro*. Truncation of the MPPKFDP motif also reduced the poly-phenylalanine synthesis in both hybrid ribosome and yeast mutagenesis studies. In addition, G→A/P substitutions to the conserved GPLG motif of helix-1 reduced poly-phenylalanine synthesis to 9–32% in yeast ribosomes. We propose that the flexible N-terminal residues of uL11, which could extend up to ∼25 Å from the N-terminal domain of uL11, can form transient interactions with the uL10 that help to fetch and fix it into a position ready for recruiting the incoming translation factors and facilitate protein synthesis.

## INTRODUCTION

Ribosomal protein uL11 and the lateral stalk constitute the GTPase-associated center of ribosomes where translation factors are recruited and activated (1,2). Eukaryotic ribosomes can use translation factors eEF1α and eEF2 only from eukaryotes but not EF-Tu and EF-G from bacteria. Domain-specific recognition of eukaryotic translation factors is mediated mainly by the lateral stalk P-complex (3). However, eukaryotic ribosomal protein uL11 (formerly eL12) is involved in domain-specific action of the factors in translation elongation (3). Eukaryotic P-complex is consisted of two copies of P1/P2 heterodimers binding to uL10 (formerly P0) to form a pentameric complex of uL10(P1/P2)_2_ (4,5). The topological arrangement of the P-complex was characterized by NMR spectroscopy and biochemical analyses (6,7). In bacterial ribosomes, the lateral stalk complex is consisted of two or three copies of bL12 homodimers binding to uL10 (8,9). Replacing the uL11 and the uL10 (bL12•bL12)_2_ stalk complex of *Escherichia coli* ribosomes with eukaryotic uL11 and P-complex resulting in hybrid ribosomes that can use the eukaryotic eEF1 and eEF2 for translation elongation (3). Moreover, the hybrid ribosomes were insensitive to thiostrepton, an antibiotic that inhibits prokaryotic ribosomes (3). On the other hand, substitution of bacterial uL11 into yeast ribosomes rendered them to be sensitive to antibiotic thiostrepton (10). Similarly, replacing the stalks of *E. coli* ribosomes with archaeal uL11 and P-complex resulting in hybrid ribosomes that can use archaeal translation GTPases (11–13).

Both the bacterial lateral stalk and the archaeal/eukaryotic P-complex play an important role in recruiting translation factors to the ribosome. In bacteria, lateral stalk binds multiple copies of EF-Tu–aminoacyl–tRNA-GTP ternary complexes to create a high local concentration of translation factors and delivers them to the GTPase-associated center rapidly and accurately (14). Recently, this suggestion is further supported by high-speed atomic force microscopy, which shows that the P-complex collects archaeal translation factors aEF1α and aEF2 to enrich their local concentration near archaeal ribosomes (15,16). Deletion of both copies of *RPL12A* and *RPL12B* genes encoding uL11 in yeast resulting in significant decreases in both cell growth rate and ribosome translation activity (17,18), which could be rescued by transformation with an intact copy of the uL11 gene (17). The absence of uL11 also caused a reduction of eEF1α/eEF2-dependent polyphenylalanine synthesis activity in hybrid ribosomes (3). On the other hand, it was reported that knocking-out of uL11 in yeast increased misincorporation and readthrough rate in protein translation (19).

Eukaryotic uL11 is also required for the biogenesis of ribosomes. The structure of ribosome-bound eukaryotic uL11 is well characterized (20). The mammalian uL11 contains a C-terminal domain (CTD) responsible for binding to the 28S rRNA and an N-terminal domain (NTD) that makes contacts with translation factors (2,21). In uL11-deficient yeast ribosomes, the amount of bound acidic proteins P1α/P2β is drastically reduced (17). 43S pre-initiation complex was present in uL11-deficient yeast, indicating that 60S ribosome maturation was disturbed (18,22–23). Pre-rRNA processing analysis of uL11-defective yeast cells shows that 27S pre-rRNA, 7S pre-rRNA intermediates and the 25S/18S ratio decrease significantly, causing the 60S subunits to not mature properly due to the premature degradation of rRNAs (22,24). Moreover, uL11 and uL10 help each other to bind ribosome. uL10 can be washed out from uL11-deficient yeast ribosomes by 0.5 M NH_4_Cl buffer while 1 M NH_4_Cl buffer was needed for wild-type ribosomes, showing that uL10 has a higher affinity to ribosome in the presence of uL11 (17). In addition, binding of yeast uL11 to ribosomes was decreased in the Mrt4/uL10 chimera ribosomes in which the first 137 residues of uL10 are replaced by residues of Mrt4 (25).

Knocking out uL11 makes yeast sensitive to antibiotics targeting the aminoacyl-tRNA site (A-site). uL11-deficient yeast is hypersensitive to aminoglycoside antibiotic paromomycin (22), which binds to ribosomal RNA in the A-site (26). A similar effect was observed for aminoglycoside antibiotic hygromycin B (22), an inhibitor preventing the movement of the tRNA from the A-site to the P-site (27). uL11-deficient cells exhibited increase sensitivity toward sordarin (22), which inhibits translocation of yeast eEF2 (28) to carry out its function at the A-site (29). In contrast, cycloheximide, which inhibits elongation through binding to the E-site of the 60S ribosomal unit (30), has no significant effect on uL11-deficient cells (22).

Although eukaryotic, archaeal and bacterial uL11 are structurally homologous, eukaryotic uL11 contains a highly conserved motif, MPPKFDP, that is not found in archaeal and bacteria uL11. Interestingly, these N-terminal residues are disordered in the structures of ribosome-bound uL11. To understand the role of the N-terminal residues of uL11, we determined the solution structure and characterized the backbone dynamics of human uL11 by NMR spectroscopy, and showed that the N-terminal residues of uL11 are unstructured and flexible in solution. We further showed that uL11 can interact with uL10(P1/P2)_2_*in vitro*. Truncating the MPPKFDP motif of uL11 abolished the interaction with uL10(P1/P2)_2_ and reduced poly-phenylalanine synthesis in hybrid ribosomes and yeast ribosomes. Based on our results, a model of how uL11 could facilitate protein synthesis is proposed.

## MATERIALS AND METHODS

### Plasmid construction

#### E. coli expression

Coding DNA sequences of human ribosomal stalk proteins uL10, P1 and P2 were amplified by PCR and cloned into expression vectors as pRSETA-His-uL10, pET8c-P1 and pET8c-P2 as previously described (6,31). Coding DNA sequences of human ribosomal protein uL11 (32) was amplified by PCR and cloned into expression vector pRSETA-uL11. The truncation mutant lacking the N-terminal seven amino acids of uL11 (uL11 ΔN7) was created using the QuikChange site-directed mutagenesis kit (Agilent) according to the manufacturer's instructions.

#### S. cerevisiae expression

To construct the balancing plasmid (pRPL12B-myc), a PCR fragment containing the DNA sequence of *Saccharomyces cerevisiae* uL11 with a C-terminal c-Myc tag flanked by the alcohol dehydrogenase 1 (ADH1) promoter and the ADH1 terminator was generated by overlap extension PCR and cloned into pRS416 (33) with a BamHI site and an XhoI site as previously described (34). The DNA sequence of uL11 was amplified from yeast genomic DNA. DNA sequences for the ADH1 promoter and ADH1 terminator were amplified from pGADT7 (TaKaRa). For constructing pRPL12B-T7, i.e. the plasmid remaining in the uL11 knockout strain after 5′-fluoroorotic acid (5′FOA) counter selection, PCR fragment that contains uL11 gene with C-terminal T7 tag and flanked by ADH1 promoter and terminator was cloned into pRS415 (33) with a BamHI site and an Xh*o*I site. Plasmids containing mutant uL11 were constructed by site-directed mutagenesis on the coding sequence of uL11 in pRPL12B-T7. The plasmids used are listed in Supplementary Table S1.

### NMR sample preparation

*Escherichia coli* strain soluBL21 (Genlantis) was transformed with the pRSETA-uL11. The transformed strain was first cultured in 20 ml Luria-Bertani broth overnight at 37°C. The cells were harvested and resuspended in M9 medium (6 g/l Na_2_HPO_4_, 3 g/l KH_2_PO_4_, 0.5 g/l NaCl, and 2 mM MgSO_4_) containing 4 g/l [^13^C]glucose, 1 g/l [^15^N]ammonium chloride, 1× vitamin mix (4.1 μM biotin, 7.2 μM choline chloride, 4.2 μM calcium pantothenate, 2.3 μM folic acid, 11.1 μM myo-inositol, 8.2 μM nicotinamide, 4.9 μM pyridoxal HCl, 0.3 μM riboflavin, 3.0 μM thiamine HCl), 1× metal mix (50 μM FeCl_3_, 20 μM CaCl_2_, 10 μM MnCl_2_, 10 μM ZnSO_4_, 2 μM CoCl_2_, 2 μM CuCl_2_, 2 μM NiCl_2_) and 100 μg/ml ampicillin. The culture was then incubated at 37°C until the OD_600_ reached 1.0. Then expression was induced for 16 h at 25°C with 0.4 mM isopropyl β-d-1-thiogalactopyranoside (IPTG). One liter of cell pellet was resuspended with 30 ml of buffer A (20 mM sodium phosphate, pH 7.4) and lysed by sonication. The filtered supernatant of the cell lysate was loaded onto a 5 ml HiTrap SP HP column (GE Healthcare) pre-equilibrated with buffer A. After being extensively washed with buffer A, uL11 was eluted with a linear NaCl gradient of 200 ml from 0 M to 0.5 M. uL11 was eluted as a single peak at about 0.3 M NaCl. The fractions collected were dialysed against 20 mM sodium phosphate, 0.05 M Na_2_SO_4_, 5 mM DTT, 0.3% CHAPS, pH 7.4. Protein was concentrated to 5 ml and loaded onto a HiLoad Superdex 75 26/600 gel filtration column (GE Healthcare) pre-equilibrated with 20 mM sodium phosphate, 0.05 M Na_2_SO_4_, 5 mM DTT, 0.3% CHAPS, pH 7.4. uL11 was eluted at about 200 ml. The protein samples were concentrated to 0.3 mM for NMR experiments.

### Structure determination

All NMR experiments were carried out using 0.3 mM uL11 containing 5%(v/v) D_2_O at 306K using Bruker Avance 700 MHz spectrometers. The sequential backbone assignments were obtained from four triple-resonance experiments: HNCACB (35) (36), CBCA(CO)NH (37), HNCA (38,39) and HN(CO)CA (38,39). The side chain resonances were assigned by seven spectra: TOCSY-HSQC (40,41), NOESY-HSQC (41,42), HCCH-COSY (43), HCCH-TOCSY (43,44), H(CC)(CO)NH (45), (H)CC(CO)NH (45), HBHA(CO)NH (36,46). Stereospecific assignments for the methyl groups of valine and leucine were obtained using a 10% ^13^C-labeled sample (47). Inter-proton distance restraint information was obtained from the following NOESY-type experiments: 3D ^1^H,^15^N-NOESY-HSQC (41,48), 3D ^1^H, ^13^C-NOESY-HSQC (42), 3D ^1^H,^13^C-HSQC-NOESY-^1^H,^13^C-HSQC (49,50) and 2D ^1^H–^1^H NOESY (51). Chemical shifts were referenced with respect to 4,4-dimethyl-4-silapentane-1-sulfonate (DSS). All multidimensional NMR spectra were processed with NMRpipe (52) and analysed by NMRViewJ (53). Dihedral angle restraints were derived from the TALOS-N program (54). Hydrogen bond restraints, derived from hydrogen-deuterium exchange experiments, were included for secondary structure elements. NOEs were assigned with the help of ARIA 2.3 (55) and CNS 1.2 (56,57). Final structural refinement was performed using Xplor-NIH 3.3 (58). 10 best structures with the lowest total energy and no restraint violation were selected.

### ^15^N relaxation experiments

^15^N-labeled samples of uL11 were used to determine the ^15^N longitudinal relaxation rates (*R*_1_), transverse relaxation rates (*R*_2_), and heteronuclear NOEs using Bruker Avance 700 MHz spectrometers at 306 K. Relaxation delays for measuring *R*_1_ were 0.002, 0.07, 0.128, 0.267, 0.533, 0.800, 1.120, 1.440 and 1.867 s and for measuring *R*_2_ were 0.017, 0.034, 0.051, 0.068, 0.085, 0.102, 0.136 and 0.204 s. *R*_1_ and *R*_2_ rates were obtained by fitting peak intensities to an exponential decay using NMRView (53). The standard deviations for the *R*_1_ and *R*_2_ relaxation rates were obtained by Monte Carlo simulation implemented in NMRView. The steady-state heteronuclear ^15^N{^1^H} NOEs were measured in spectra acquired with and without ^1^H presaturation (with a series of high-power 120° pulses of 18 μs each) in an interleaved manner (59,60) and were defined using the term (*I* – *I*o)/*I*o, where *I* and *I*o are peak intensities measured with and without presaturation, respectively. For *R*_1_ and *R*_2_ experiments, a recycle delay of 3 s was used between transients while 5 s was used for heteronuclear ^1^H–^15^N NOEs. The NMR spectra for relaxation experiments were processed with NMRPipe (52). Uncertainties in intensity measurements were estimated from the root-mean-square noise of the spectra. Effective correlation times of the backbone NH bonds were calculated using τ_c(eff)_ = 5/2 *J*(0)_eff_, where *J*(0)_eff_ is the effective spectral density at zero frequency calculated from *R*_1_, *R*_2_ and ^15^N{^1^H} NOE as described (61,62).

### Hybrid ribosome preparation and functional assays

Bacterial 50S ribosomal subunit was extracted from L11-deficient *E. coli* strain and the ribosomal stalk proteins uL10 and bL12 were released by washing with 50% ethanol, 0.5 M NH_4_Cl as previously described (3). Human ribosomal stalk proteins uL10, P1 and P2 were purified as previously described (6,31). P-complex was prepared using *in vitro* reconstitution as previously described (6). Human uL11 and mutants were expressed at 25°C overnight in *E. coli* strain SoluBL21(DE3) from Genlantis. The supernatant was collected after cell lysis in 20 mM phosphate buffer at pH 7.4 and loaded to an SP FastFlow column (GE Healthcare) pre-equilibrated with the same buffer. After extensive washing, a 200 ml gradient from 0 to 0.5 M NaCl was applied. Fractions containing uL11 were eluted at around 0.3 M NaCl and pooled and concentrated to a final volume of 5 ml with the maximum concentration of 10 mg/ml. The protein sample was then loaded to a HiLoad Superdex 75 26/600 gel filtration column pre-equilibrated with buffer containing 20 mM phosphate buffer, 0.15 M NaCl at pH 7.4, and was eluted as a single peak. The hybrid 50S particle was formed by mixing the *E. coli* 50S core with the human P-complex and uL11, using the same protocol for preparing *B. mori–E. coli* hybrid ribosome as described in (63). Bacterial 30S ribosomal subunit was extracted from *E. coli* Q13 as previously described (3). Purification of elongation factors was described previously (64). The eEF-1α/eEF-2-dependent poly-phenylalanine synthesis of *Homo sapiens–E. coli* hybrid ribosome was assayed as described previously (63,65).

### *In vitro* NHS pull-down assay

Protein samples of uL11 or uL11ΔN7 were dialyzed against coupling buffer with 0.2 M NaHCO_3_, 0.5 M NaCl at pH 8.3. For every 200 μl *N*-hydroxysuccinimide (NHS)-activated Sepharose resin pre-activated with 1 mM HCl, 1.5 mg of protein was coupled at 4°C overnight. The resin was washed with Tris buffer containing 0.1 M Tris, 0.5 M NaCl at pH 8.5, and then with acetate buffer containing 0.1 M acetate, 0.5 M NaCl at pH 4.5. After three rounds of alternate washing with Tris buffer and acetate buffer, the resin was equilibrated with a buffer containing 20 mM Tris at pH 7.8. 60 μM of purified samples of P-complex were then incubated with the resin at 4°C overnight. After extensive washing with the equilibration buffer (20 mM Tris, pH 7.8), the bound proteins were eluted with buffer containing 20 mM Tris, 1 M NaCl at pH 7.8. NHS-activated Sepharose resin coupled with glycine was included as the negative control. Results were analyzed with SDS-PAGE or western blot using home-raised anti-P antibody and anti-uL10-EXT antibody. Anti-P polyclonal rabbit antibody was raised against the peptide, KEESEESDDDMGFGLFD, that is conserved in uL10, P1 and P2. uL10-EXT polyclonal rabbit antibody were obtained against the peptide, NRVAAPARAGAVAPC, that is conserved in the EXT domain of uL10.

### Creation of yeast mutant strains

In *S. cerevisiae*, there are two paralogs of uL11, *RPL12A* and *RPL12B*. To facilitate the knockout, the *RPL12B*-knockout strain, Y04254, was used as the parental strain. pRPL12B-myc was transformed into strain Y04254 to yield strain KBeL1. The *RPL12A* gene was replaced by the natRMX4 selection marker gene (66) by homologous recombination using the microhomology PCR-mediated targeting technique as previously described (34), to yield strain KBeL2. Then pRP12B-T7 or its mutant plasmids expressing wild-type uL11 or mutant uL11 was transformed into the strain KBeL2 to yield strains (KBeL3-WT, KBeL3-ΔN7, KBeL3-G33A, KBeL3-G33P, KBeL3-G36A, KBeL3-G36P). The balancing plasmid (pRPL12B-myc) was removed by growing the KBeL3 yeast strain on agar plate containing 5′FOA. Every step and genetic manipulation and the counterselection results were verified by western blot analysis, using anti-T7 (Abcam), anti-c-Myc (Genscript) and home-raised anti-uL11 antibody. Anti-uL11 polyclonal rabbit antibody was raised against the peptide, PRDRKKQKNIKHSG, that is conserved in uL11. All yeast strains were grown at 30°C in yeast extract adenine dextrose medium or synthetic dextrose (SD) medium lacking uracil and/or leucine. The yeast strains used in this study are listed in Supplementary Table S2.

### Purification of ribosomes

One liter of yeast culture was grown to an OD_600_ of 0.6–1.0 and collected by centrifugation at 10 000 g, 4°C for 10 min. The pellet was then washed with buffer A (20 mM HEPES, 100 mM potassium acetate, 2 mM magnesium acetate, and 5 mM dithiothreitol, pH 7.4) and lysed by JN-mini low-temperature ultra-high pressure continuous flow cell disruptor (JNBIO) at 1800 bar, 4°C. The crude lysate was centrifuged at 10 000 g, 4°C for 30 min twice to remove cell debris. The supernatant, also known as S30, was collected and subjected to high-speed centrifugation at 184 000 g, 4°C for 4 h in a Type 70 Ti rotor (Beckman Coulter). The supernatant collected was then subjected to 30–70% ammonium sulfate precipitation and centrifuged at 13 000 g, 4°C for 2 min. After centrifugation, the supernatant, also known as S100, was collected. It served as the source of supernatant factors in the polyphenylalanine synthesis assay. The pellet was re-suspended with buffer B (20 mM HEPES, 100 mM potassium acetate, 12 mM magnesium acetate, and 5 mM dithiothreitol, pH 7.4) and centrifuged in a 33% sucrose cushion (in buffer B) at 184 000 g, 4°C for 4 hours in a Type 70 Ti rotor, to get crude ribosome. The crude ribosome was re-suspended with buffer B and then centrifuged in 7–47% sucrose gradient in an SW32Ti rotor (Beckman Coulter) at 100 000 g, 4°C for 16 h. Proper 80S fractions with OD_260_/OD_280_ ratio of 2:1 were collected and centrifuged again at 184 000 g, 4°C for 4 h in a Type 70Ti rotor to collect the 80S ribosomes pellet. The pelleted 80S ribosomes were resuspended with buffer B and quantified by OD_260_ as described by Matasova *et al.* (67). Purified ribosomes were checked by Western blot analysis using S6, uL10 and GAPDH antibodies (GenScript).

### Polyphenylalanine synthesis assay

The reaction mixture of 17.5 μl was prepared by mixing 80S ribosomes (0.136 μM), S100 fraction (OD_280_ = 0.1), 20 mM HEPES (pH 7.4), 100 mM potassium acetate (Sigma-Aldrich), 12 mM magnesium acetate (Sigma-Aldrich), 0.05 mM spermine (Sigma-Aldrich), 20μM spermidine (Sigma-Aldrich), 7.5 mM creatine phosphate (Sigma-Aldrich), 0.0432 mg/ml of creatine kinase (Sigma-Aldrich), 0.1 mM GTP (Sigma-Aldrich), 0.2 mM ATP (Sigma-Aldrich), 0.5 mM tRNA (Sigma-Aldrich), 0.5 mM calcium chloride (Affymetrix), 12.5 units of RNAsin plus (Promega), 12.5 units of micrococcal nuclease (New England Biolabs). After digestion of endogenous mRNA for 15 min at 20°C, 1 μl of 37.5 mM EGTA (Sigma-Aldrich) was added to the above reaction mixture to inactivate the micrococcal nuclease. 2.5 μl of 2 mg/μL poly-U RNA (Santa Cruz Biotechnology) and 4 μL of 1 mCi/ml [^3^H]-l-phenylalanine (PerkinElmer) were added to the reaction mixture and incubated for 30 min at 30°C. The reaction was quenched by adding 25% trichloroacetic acid (Sigma-Aldrich) and incubated on ice for 1 h. The precipitate was collected on a GF/B grade glass microfiber filter paper (Whatman). After the filter was washed three times with 8% trichloroacetic acid and a final wash with acetone, the amount of radioactively labelled polyphenylalanine was quantified using an LS6500 liquid scintillation counter (Beckman Coulter). One-way ANOVA was performed using the program PRISM (GraphPad Software, LLC) to analyse the statistical difference between the wild-type ribosome and the mutant ribosome.

### Molecular dynamics (MD) simulation

MD simulation was performed for the ribosomal stalk base [i.e. uL10, uL11 and H42-H44 stem loops (residues 1995–2048) of 28S rRNA]. Starting coordinates were obtained from the structure of mammalian ribosomes in complex with eRF1/ABCE1 (PDB: 5LZV). Residues of helix-6 of uL11 were refitted to the cryo-EM densities of 5LZV with reference to the crystal structure of *Methanococcus jannaschii* uL11 (68) (PDB: 5DAR) and refined using the program PHENIX (69) (Supplementary Figure S4). The starting coordinates of the N-terminal residue 2–8 of uL11 were derived from two conformers of the ensembles of NMR structure determined in this study (Supplementary Figure S4C). The protonation states of amino acid residues were predicted using the web server H++ (http://newbiophysics.cs.vt.edu/H++/) (70). The starting structures of two independent simulations, MD1 and MD2, are included in the supplementary data. MD simulation was performed using the program GROMACS 2021.3 (71) using the amber99sb force field (72) with modified parameters for potassium and chloride ions (73). The structure was solvated with SPC/E waters, 7 mM MgCl_2_ and 150 mM KCl in a dodecahedron box with a minimum distance of 1.1 nm from the box boundaries. Non-bonded interactions were calculated with a cut-off distance of 1.0 nm. Long-range electrostatic interactions were calculated by particle-mesh Ewald summation with a 0.12 nm grid and an interpolation order of 4. Covalent bonds were restrained using the LINCS algorithm (74). The system was minimized and then equilibrated for 1 ns of constant NVT simulation and 1 ns of constant NPT simulation with position restraints on protein and RNA. The system was coupled with a Berendsen thermostat at 300 K with a time constant of 0.1 ps and a Parrinello-Rahman barostat at 1 bar with a time constant of 1 ps. A production 500 ns MD trajectory was simulated in an NPT ensemble for each of the starting structures. Integration was performed using the Verlet scheme with a 2 fs time step.

### Antibiotic sensitivity test

Antibiotic sensitivity test was performed as described previously (22). In brief, overnight culture of yeast cells were serially diluted to OD_600_ = 0.1, 0.01, 0.001, 0.0001. 3 μl of diluted yeast culture was spotted onto SD(-Leu) agar plate supplemented with 4 μg/ml sordarin, 20 ng/ml cycloheximide, 20 μg/ml hygromycin B and 500 μg/ml paromomycin. Growth on agar plates without any antibiotics was treated as positive controls. The cells were grown at 30°C for 3 days.

### Measurement of growth rates of yeast strains

Yeast cells were inoculated at OD_600_= 0.1 in 24-well culture plates containing 800 μl SD medium lacking leucine. The yeast suspension culture was incubated at 25°C, 30°C or 35°C in CLARIOstar microplate reader (BMG Labtech) with double orbital shaking at 300 rpm. OD_600_ was measured every 15 min using orbital averaging scan mode with a scan diameter of 10 mm. The yeast growth rate was calculated by fitting the data to the exponential growth equation as described previously (75).

## RESULTS

### uL11 undergoes major conformational changes upon binding to ribosomes

**Figure 1.**
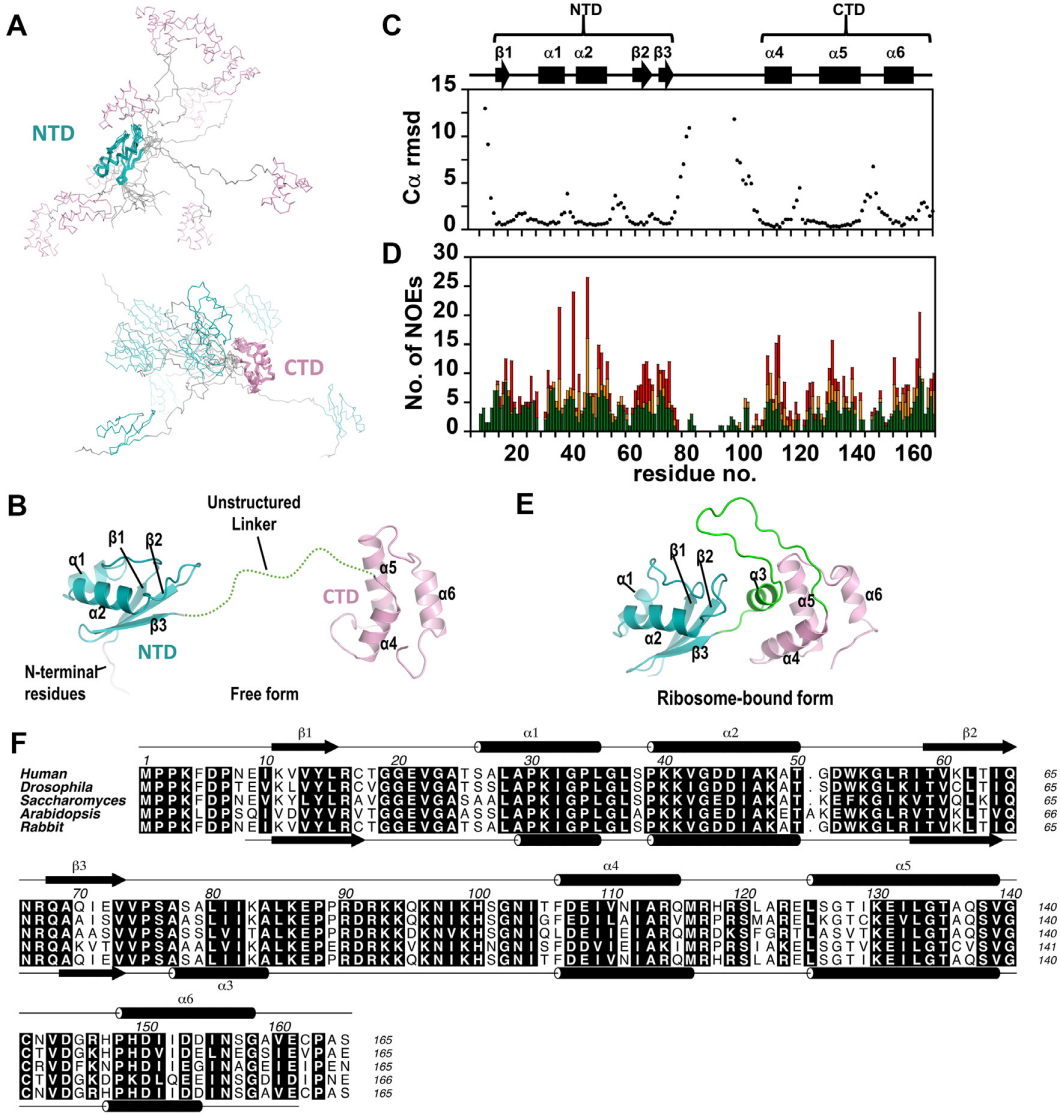
Solution structure of human uL11 consists of NTD and CTD linked with an unstructured linker region. (**A**) An ensemble of the 10 best structures was superimposed with residues in the NTD (residue 10–73, teal, upper panel) and in the CTD (residue 106–165, pink, lower panel). (**B**) A cartoon representation of the solution structure of uL11. The NTD contains two helices (α1, α2) packed against an anti-parallel β-sheet with three strands (β1, β2, β3), while the CTD contains three helices (α4, α5, α6). (**C**) Cα RMSD per residues. The secondary structure elements of the NTD and CTD are indicated. (**D**) Number of sequential (|*i* – *j*| = 1, green), medium (|*i* – *j*| = 2–4, orange) and long (|*i* – *j*| > 4, red) range NOEs along the primary sequence. (**E**) Ribosome-bound form of rabbit uL11 (PDB: 6MTD). It is noted that helix (α3) that is present in ribosome-bound uL11 becomes unstructured in the free form of uL11. The N-terminal residues (1–9) are disordered in both free and bound forms of uL11. (**F**) Sequence alignment of eukaryotic uL11. Secondary structure elements of the solution structure of human uL11 and ribosome-bound rabbit uL11 (PDB: 6MTD) are shown above and below the alignment respectively. Molecular graphics were created using the program PyMOL.

We have determined the solution structure of human uL11 by NMR spectroscopy and the statistics of the structural calculation are summarized in Table 1. The ensemble of the 10 best structures is shown (Figure 1A). The solution structure of uL11 folds into an NTD and a CTD separated by a linker region (Figure 1B). Judged by the Cα root-mean-square displacement (Figure 1C), the NTD is defined from residue 10 to 73 and the CTD from residue 106 to 165. There were no distance restraints between the NTD and CTD, suggesting that the two domains fold independently. The linker region between the NTD and CTD lacks long-range (|*i* – *j*| > 4) distance restraints and is unstructured (Figure 1D). In contrast, when uL11 is bound to ribosome, the linker region folds into helix 3 that forms hydrophobic interactions with both NTD and CTD (Figure 1E and F). The lack of helix 3 in the free form results in a structural rearrangement in helices 4, 5 and 6 of the CTD (Figure 1B). On the other hand, the NTD is structurally similar between the free and bound forms. Taken together, our results suggest that the linker region is intrinsically disordered that only becomes structured when bound to the ribosomes.

**Table 1.** Structural calculation statistics

**Distance and dihedral restraints**	
Total number of NOE	853
Unambiguous	705
intra-residue (|*i* – *j*| = 0)	345
sequential (|*i* – *j*| = 1)	214
medium-range (1<|*i* – *j*| < 5)	62
long-range (|*i* – *j*| > 4)	84
ambiguous	148
Number of hydrogen bond restraints	94
Number of dihedral angle restraints	144
**Structure statistics**	
*Violations*	
Distance restraints (Å)	0.059 ± 0.004
Dihedral restraints (°)	0.32 ± 0.11
No. of dihedral angles with violation >5°	0
No. of distant restraints with violation >0.5 Å	0
*Deviation from idealized geometry*	
Bond length (Å)	0.0071 ± 0.0003
Bond angle (°)	0.86 ± 0.01
Improper (°)	0.56 ± 0.02
*Average RMSD to mean structure (Å)* ^a^	
backbone	0.605 Å (NTD), 0.477 Å (CTD)
heavy	0.995 Å (NTD), 0.898 Å (CTD)
*Average Ramachandran plot statistics (%)*	
Most favoured	83.1
Additional allowed	14.5
Generously allowed	2.4
Disallowed	0

^a^RMSD of secondary structures of NTD and CTD were calculated.

### The flexible and conserved N-terminal residues of uL11 are involved in interacting with the P-complex *in vitro*

**Figure 2.**
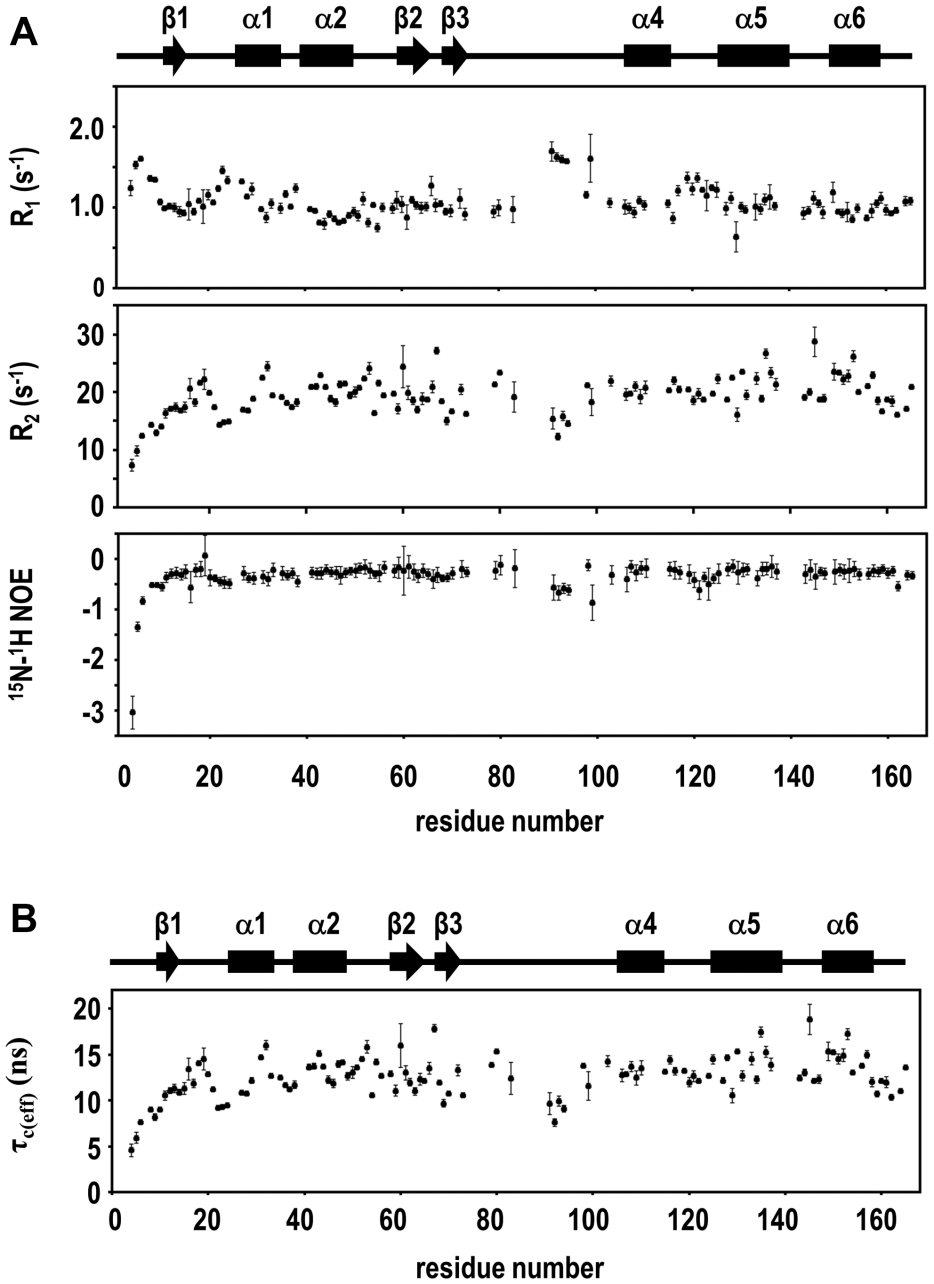
N-terminal residues of uL11 are flexible. (**A**) Longitudinal relaxation rates (*R*_1_), transverse relaxation rates (*R*_2_), and heteronuclear nuclear Overhauser enhancement (^15^N{^1^H} NOE) were obtained from ^15^N relaxation experiments and plotted against the residue number. Values of the mean and standard deviation of *R*_1_, *R*_2_ and ^1^H–^15^N NOE were 1.1 ± 0.2 s^−1^, 19 ± 3 s^−1^, and −0.34 ± 0.30 respectively. (**B**) τ_c(eff)_ was estimated from *R*_1_, *R*_2_ and ^15^N{^1^H} NOE using the reduced spectral density mapping approach and are plotted against the residue number. Secondary structure elements of uL11 were shown above the graphs.

The backbone dynamics of uL11 were characterized by ^15^N–^1^H longitudinal (R_1_) and transverse (R_2_) relaxation rates and ^15^N{^1^H} heteronuclear NOE (Figure 2A). The average values of *R*_1_, *R*_2_ and ^15^N{^1^H} NOE were 1.1 ± 0.2 s^–1^, 19 ± 3 s^–1^ and −0.34 ± 0.30, respectively. Effective correlation times, τ_c(eff)_, for the reorientation of the backbone NH bonds were estimated from *R*_1_, *R*_2_ and ^15^N{^1^H} NOE using the reduced spectral density mapping approach (Figure 2B) (61,62). Residues 4–6 have fast τ_c(eff)_ (<8 ns) and large negative values of ^15^N{^1^H} NOE (<−0.8) (Figure 2B) and lack long-range distance restraints, suggesting these N-terminal residues of uL11 are flexible and disordered in the free form. Coincidentally, these N-terminal residues are not defined and are likely disordered in ribosome-bound uL11 (Supplementary Figure S1).

**Figure 3.**
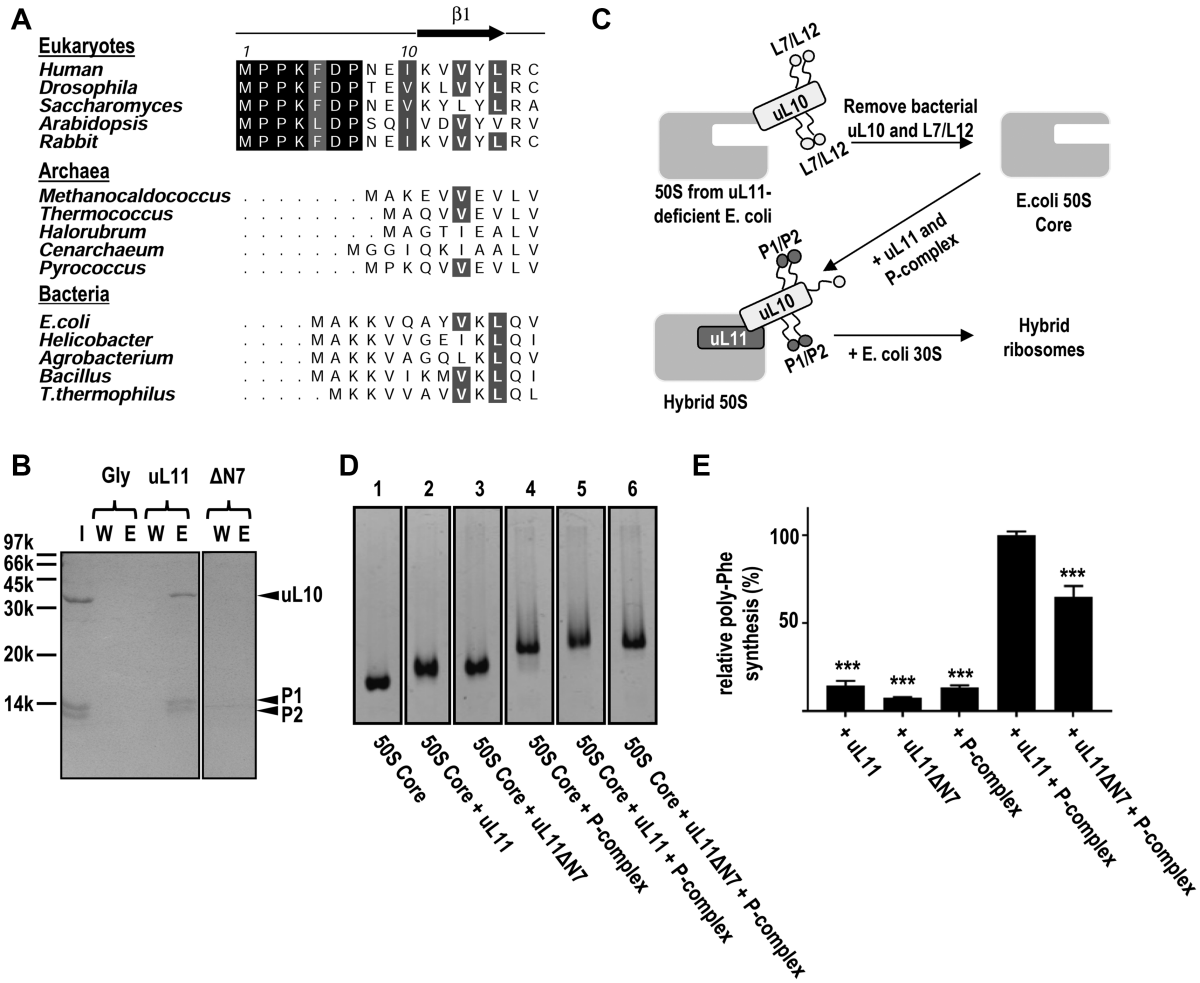
The conserved N-terminal seven residues of uL11 are important for interacting with the P-complex *in vitro* and protein synthesis in hybrid ribosomes. (**A**) Multiple sequence alignments of N-terminal residues of uL11 in eukaryotes, archaea and bacteria. The first seven residues of uL11 are conversed in eukaryotes but not in archaea and bacteria. (**B**) Truncation of the conserved N-terminal residues of uL11 abolished its interaction with P-complex uL10(P1/P2)_2_. Purified protein samples of uL10(P1/P2)_2_ (lane I) were loaded to NHS-resin coupled with glycine, uL11 or uL11ΔN7. After extensive washing to ensure all unbound proteins were removed in the last wash fractions (lanes W), bound proteins were eluted by 1 M NaCl, 20 mM Tris, pH 7.8 (lanes E). Our results showed that the P-complex uL10(P1/P2)_2_ was pulled down by uL11 but not by uL11ΔN7, suggesting that the N-terminal residues of uL11 are important for the interaction between uL11 and the P-complex uL10(P1/P2)_2_ (**C**) The schematic diagram of the preparation of hybrid ribosomes. Bacterial 50S ribosomal subunit was extracted from L11-deficient *E. coli* strain (AM68) and the bacterial ribosomal stalk proteins uL10 and bL12 were washed out with 50% ethanol and 0.5 M NH_4_Cl to obtain the 50S core ribosomes, which was then mixed with purified samples of human P-complex uL10(P1/P2)_2_ and uL11 to form the 50S hybrid ribosome particles. Bacterial 30S ribosomal subunit was added to assemble the hybrid ribosomes for the *in vitro* translation assay. (**D**) Examination of various hybrid 50S particles by electrophoretic mobility shift assay. L11-deficient *E. coli* 50S core (lane 1) was incubated with uL11 (lane 2), uL11ΔN7 (lane 3), P-complex uL10(P1/P2)_2_ (lane 4), P-complex with uL11 (lane 5) and P-complex with uL11ΔN7 (lane 6). The formations of different hybrid ribosome particles were justified by upshift of bands in lane 5–6 relative to lane 4. (**E**) Different hybrid ribosome particles were assayed for poly-phenylalanine synthesis activity. The poly-phenylalanine synthesis activities are plotted as a percentage of the hybrid ribosome reconstituted with wild-type uL11 and P-complex. Hybrid ribosomes reconstituted with uL11ΔN7 and P-complex resulted in a significant decrease (to 65 ± 16% of wild-type uL11) in the poly-phenylalanine synthesis activity. *** indicates significant difference (*P* < 0.001, one-way ANOVA) of relative poly-phenylalanine synthesis when compared to the value for hybrid ribosomes reconstituted with wild-type uL11 and P-complex. Error bars represent the mean ± s.e.m. of the data from at least three measurements.

Intriguingly, eukaryotic uL11 contains a unique N-terminal extension of conserved residues (MPPKFDP), which are absent in archaeal and bacterial uL11 (Figure 3A). In eukaryotic ribosomes, uL11 and uL10 constitute the base of the ribosomal lateral stalk and the N-terminus of uL11 is located near the uL10 (Supplementary Figure S1). We, therefore, hypothesize that this N-terminal MPPKFDP motif of uL11 could play a role in interacting with the eukaryotic stalk P-complex (uL10(P1/P2)_2_). Our pull-down assay shows that the P-complex was eluted when loaded to NHS-resins coupled with uL11, but not with those coupled with glycine (Figure 3B), suggesting that uL11 can interact with the P-complex *in vitro*. On the other hand, when the MPPKFDP motif of uL11 was removed, the P-complex was not retained on NHS-resins coupled with the uL11ΔN7 variant (Figure 3B). To test if the hydrophobic residues of the N-terminal residues of uL11 are important for interacting with the P-complex, we created a quadruple-substituted variant (P2A/P3A/P5A/P7A), uL11-4A. Our pull-down assay shows that the interaction between uL11-4A and the P-complex was weaken *in vitro* (Supplementary Figure S2). Taken together, our results suggest that the conserved N-terminal MPPKFDP motif of uL11 are important in interacting with the P-complex.

### The N-terminal conserved residues of uL11 interact with uL10-EXT *in vitro*

In the ribosome structures, the extended protuberant (EXT) domain of uL10 is near the NTD of uL11 (Supplementary Figure S1). We, therefore, hypothesize that the N-terminal conserved residues of uL11 could interact with uL10-EXT. We have previously purified the *Bombyx mori* (silkworm) uL10-EXT domain and determined its NMR structure (34). *B. mori* uL10-EXT shares 78% sequence identity with human uL10-EXT (Supplementary Figure S3A). Here, we performed *in vitro* pull-down assay and shows that uL11 interacts with *B. mori* uL10-EXT (Supplementary Figure S3B). Such interaction was weaken when the N-terminal residues of uL11 were truncated (uL11ΔN7) or the N-terminal hydrophobic residues were substituted by alanine (uL11-4A) (Supplementary Figure S3B), suggesting the N-terminal residues of uL11 are involved in interaction with uL10-EXT.

### MD simulation of ribosomal stalk base

To explore how the N-terminal residues of uL11 may interact with uL10, we have performed MD simulation of the ribosomal stalk base consisted of uL10, uL11 and the H42-H44 stem loop of 28S rRNA. The starting structures of MD simulation were derived from the ribosome structures in complex with eRF1/ABCE1 (release factor 1 / ATP-binding cassette subfamily E member 1) (21) (Supplementary Figure S4). In this state of ribosome, the uL10-EXT is away from and making no contact with the uL11-NTD (Supplementary Figure S1). Two trajectories of 500 ns each were calculated (Supplementary Figure S5). In both simulations, uL10-EXT moved towards uL11-NTD as the distance between the two domains were shortened from ∼40 Å to ∼25 Å after ∼100 ns (Supplementary Figure S5B). In the MD1 simulation, the N-terminal residues of uL11 only make transient interactions with uL10-EXT (Supplementary Figure S5C, red line). On the other hand, the N-terminal residues of uL11 interact with uL10-EXT in the MD2 simulation as these residues of uL11 are making continuous contacts (∼2 Å) with the uL10-EXT domain (Supplementary Figure S5C, blue line). As shown in a representative snapshot at 200 ns of MD2, the N-terminal hydrophobic residues of uL11 are making hydrophobic interactions with Pro118, Val121, Ile180 and Pro182 of uL10-EXT (Supplementary Figure S5D). Interestingly, these residues are conserved in uL10 (Supplementary Figure S3A).

### Deletion of N-terminal conserved MPPKFDP motif of uL11 reduced poly-phenylalanine synthesis in hybrid ribosomes and yeast ribosomes

We used the hybrid ribosome assay to test if the N-terminal MPPKFDP motif of uL11 has a functional role in protein synthesis (Figure 3C). We have previously developed a methodology to reconstitute hybrid ribosomes, where the bacterial stalk proteins from *E. coli* ribosomes were replaced with eukaryotic counterparts (3). Such replacement of stalk proteins changes the specificity of hybrid ribosomes to use eukaryotic elongation factors in translation elongation. First, bacterial 50S ribosomal subunit was extracted from L11-deficient *E. coli* strain (AM68). After removing the bacterial stalk proteins, the *E. coli* 50S core particles (Figure 3D, lane 1) were mixed with human uL11 (wild type or ΔN7 variant) and/or the P-complex to form different hybrid 50S particles (Figure 3D, lanes 2–6). Finally, the bacterial 30S ribosomal subunit was added to reconstitute the hybrid ribosomes. The reconstituted hybrid ribosomes were then assayed for *in vitro* poly-phenylalanine synthesis (Figure 3E). Our results showed that hybrid ribosomes lacking either uL11 or the P-complex were relatively inactive, suggesting both uL11 and the P-complex are required in protein synthesis. Compared with hybrid ribosomes reconstituted with wild-type uL11 and P-complex, hybrid ribosomes reconstituted with uL11ΔN7 and P-complex exhibited only ∼65% poly-phenylalanine synthesis activity (Figure 3E), demonstrating that the conserved N-terminal MPPKFDP motif of uL11 is important for protein synthesis.

**Figure 4.**
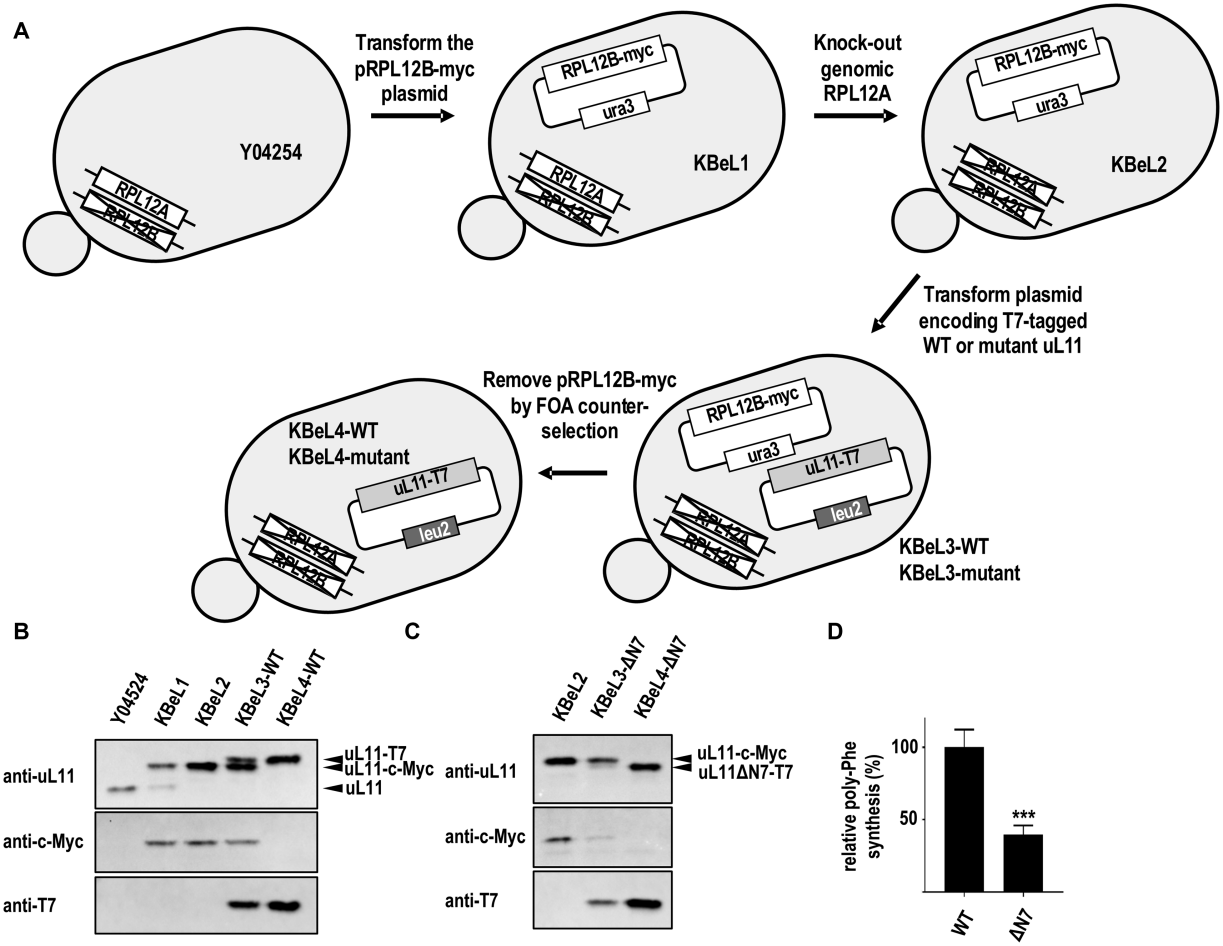
Truncation of the N-terminal residues reduced poly-phenylalanine synthesis in yeast ribosomes. (**A**) Strategy of yeast mutagenesis. RPL12B-knockout strain Y04254 was used as the parental strain. After the plasmid pRPL12B-myc was transformed into strain Y04254 to yield strain KBeL1, the RPL12A gene was knocked out by homologous recombination to yield strain KBeL2. Plasmids expressing T7-tagged wild-type or ΔN7 mutant of uL11 were transformed into the strain KBeL2 to yield KBeL3-WT and KBeL3-ΔN7 strains. The plasmid pRPL12B-myc was removed by counter-selection using 5′FOA, which is converted to the toxic compound 5-fluorouracil by *ura3*, to yield KBeL4-WT and KBeL4-ΔN7. (**B**) Cell lysates of yeast strains Y04254, KBeL1, KBeL2, KBeL3-WT and KBeL4-WT were analysed by Western blot using uL11, c-Myc, and T7 antibodies and showed that only the T7-tagged wild-type uL11 was expressed in KBeL4-WT strain. (**C**) Cell lysates of yeast strains KBeL2, KBeL3-ΔN7 and KBeL4-ΔN7 were analysed by Western blot using uL11, c-Myc, and T7 antibodies, confirming only T7-tagged uL11-ΔN7 was expressed in KBeL4-ΔN7 strain. (**D**) *In vitro* poly-phenylalanine synthesis activity on yeast ribosomes purified from KBeL4-WT and KBeL4-ΔN7 strains. The poly-phenylalanine synthesis activities are plotted as percentages of the activity of wild-type ribosomes. Yeast ribosomes with the ΔN7-uL11 variant retained only 40 ± 6% poly-phenylalanine synthesis activity, showing that the N-terminal residues of uL11 are important for protein synthesis (*t*-test; ***, *P* < 0.001). Error bars represent the mean ± s.e.m. of the data from at least three separate experiments.

Next, we asked if truncation of the N-terminal MPPKFDP motif of uL11 would affect protein synthesis in eukaryotic ribosomes. To this end, we used yeast mutagenesis to create mutant yeast strains expressing the uL11ΔN7 variant. The strategy of yeast mutagenesis is described in Figure 4A. In the yeast genome, both *RPL12A* and *RPL12B* genes encode for the uL11 ribosomal protein. The *RPL12B* knock-out strain Y04254 was used as the parental strain for yeast mutagenesis. First, the balancing plasmid pRL12B-myc expressing the c-Myc tagged wild-type uL11 was transformed into the parental strain Y04254 to create the KBeL1 strain. The endogenous *RPL12A* gene was then knocked out by homologous recombination to create the KBeL2 strain. As shown in Figure 4B, only the c-Myc tagged uL11, but not the endogenous uL11, was expressed in KBeL2 strain, suggesting that the genomic *RPL12A* gene was successfully knocked out. KBeL2 strain was transformed with plasmids expressing T7-tagged wild-type or ΔN7 variant of uL11 to create KBeL3-WT and KBeL3-ΔN7 strains. Finally, taking advantage of the fact that 5′FOA can be converted to the toxic 5-fluorouracil by orotidine 5-phosphate decarboxylase (encoded by the marker gene *ura3*), the balancing plasmid pRPL12B-myc was removed by counterselection with 5′FOA to create KBeL4-WT and KBeL4-ΔN7 strains (Figure 4A). Western blot confirmed only the T7-tagged uL11 and its ΔN7 variant were expressed in KBeL4-WT and KBeL4-ΔN7 strains, respectively (Figure 4B and C).

Yeast 80S ribosomes from KBeL4-WT and KBeL4-ΔN7 strains were purified (Supplementary Figure S6A and B) and were assayed for their *in vitro* poly-phenylalanine synthesis activity (Figure 4D). The poly-phenylalanine synthesis activity of yeast ribosomes from the KBeL4-WT strain was similar to that from the parental strain Y04254, suggesting the T7-tag on uL11 has no effect on the protein synthesis (Supplementary Figure S7). Consistent with the hybrid ribosome results (Figure 3E), truncation of the N-terminal 7 residues of uL11 reduced the poly-phenylalanine synthesis activity to ∼40% of wild-type uL11 (Figure 4D). Taken together, our results suggest that the N-terminal conserved MPPKFDP motif of uL11 is important for protein synthesis.

### The ΔN7 mutant did not affect antibiotic sensitivity and the growth rate of yeasts

It is noticed that the expression level of uL11ΔN7-T7 in KBeL3-ΔN7 strain was lower than that of c-Myc-tagged wild-type uL11 (Figure 4C). To test if the expression of uL11ΔN7-T7 is lower in the parental yeast strain, plasmids encoding T7-tagged wild-type uL11 or uL11ΔN7 were transformed to the Y04254 strain (Supplementary Figure S8A). Our results show that the expression of uL11ΔN7 was also lower than that of wild-type uL11 in the parental strain (Supplementary Figure S8B). Seemingly, the expression of uL11ΔN7 was lower when co-expressed with a wild-type uL11 (Figure 4C and Supplementary Figure S8B).

Next, we tested if the expression of uL11ΔN7 affects the sensitivity of yeast against antibiotics targeting ribosomes. Our results show that the expression of uL11ΔN7-T7 in the parental Y04254 strain or in KBeL4 did not affect the antibiotics sensitivity (Supplementary Figure S9). We also tested if the ΔN7 mutation affects yeast growth. Growth rates of KBeL4-WT and KBeL4-ΔN7 strains were measured at 25, 30 and 35°C (Supplementary Figure S10). Our results show that the growth rates of both strains were similar, suggesting truncation of the N-terminal residues of uL11 did not affect the growth of yeasts.

### Rigidifying the conserved GPLG motif reduced protein synthesis in yeast ribosomes

**Figure 5.**
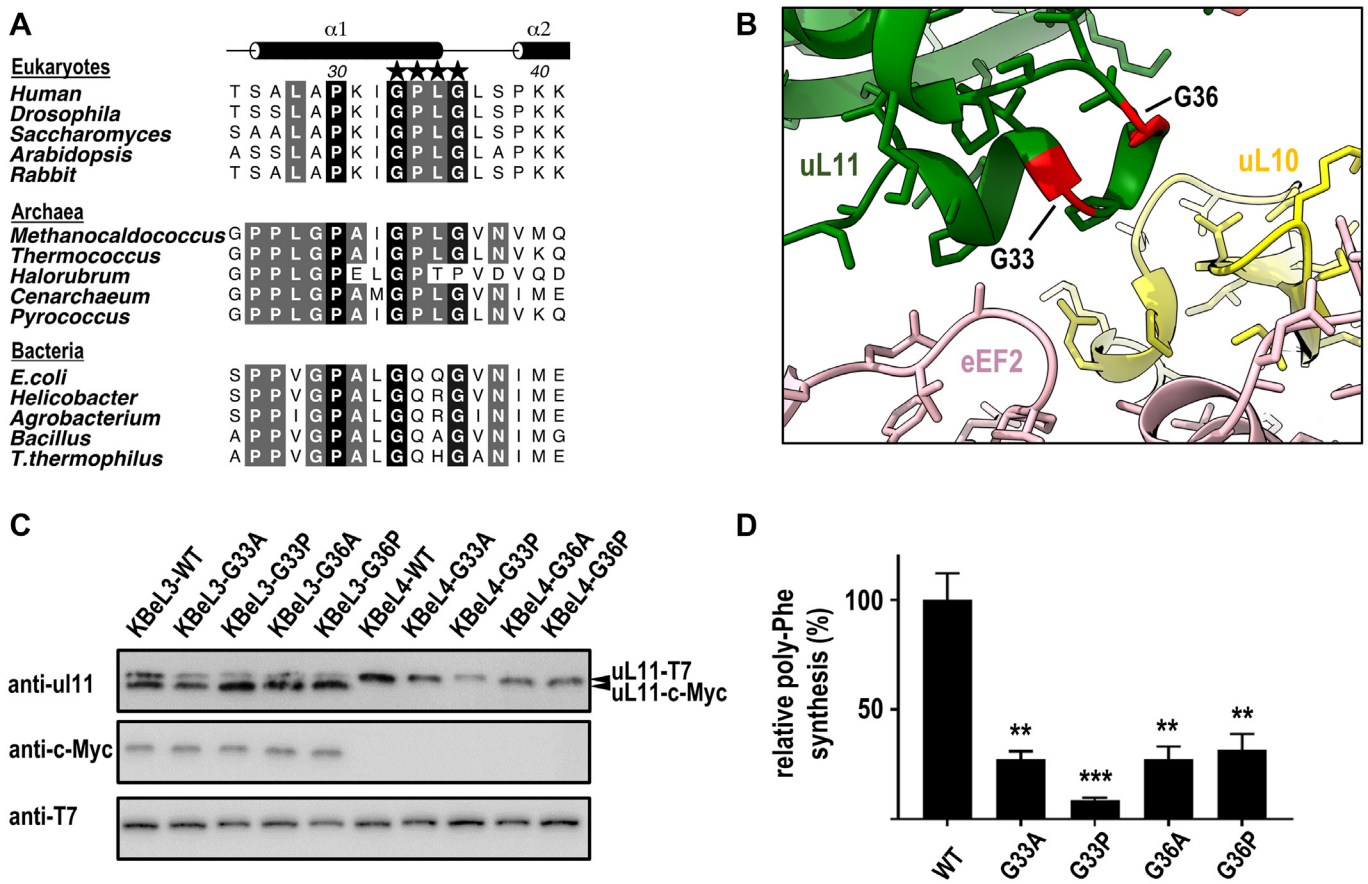
Rigidifying the GPLG motif reduced poly-phenylalanine synthesis in yeast ribosomes. (**A**) Sequence alignments of uL11. The _33_GPLG_36_ motif (indicated by asterisks) is highly conserved among eukaryotic and archaeal uL11. (**B**) The GPLG motif is located in helix-1 of uL11 (green) and is involved in binding eEF2 (pink). Cryo-EM structure of 80S ribosomes in complex with eEF2 and SERBP1 (PDB: 6MTD). The conserved Gly33 and Gly36 of uL11 are color-coded red. uL10 is color-coded yellow. (**C**) Cell lysates of wild-type and mutant yeast strains of KBeL3 and KBeL4 (after 5′FOA counter selection) were analysed by Western blot using uL11, c-Myc, and T7 antibodies, showing that the pRPL12B-myc was removed by 5′FOA counter selection, and only T7-tagged uL11 was expressed in KBeL4 strains. (**D**) *In vitro* poly-phenylalanine synthesis activity on yeast ribosomes purified from KBeL4-WT, KBeL4-G33A, KBeL4-G33P, KBeL4-G36A and KBeL4-G36P strains. The poly-phenylalanine synthesis activities are plotted as percentages of the activity of the wild-type ribosomes. The poly-phenylalanine synthesis activities of G33A, G33P, G36A and G36P were significantly reduced to 27 ± 4%, 9 ± 1%, 27 ± 6%, 32 ± 7%, respectively (one-way ANOVA; **P < 0.01; ***P < 0.001). Error bars represent the mean ± s.e.m. of the data from at least three separate experiments. Molecular graphics were created using the program UCSF ChimeraX (82).

In the cryo-EM structures of ribosomes in complex with translation factors, helix-1 of uL11 is making contacts with bound translation factors (Supplementary Figure S1) (2,21,76–78). There is a _33_GPLG_36_ motif located in the helix-1 of uL11 that is conserved in eukaryotes and archaea (Figure 5A). The GPLG motif is better defined when helix-1 makes contact with the bound eEF2 (Figure 5B and Supplementary Figure S11A) but becomes more disordered when the ribosomes are bound with aminoacyl-tRNA and eEF1α (Supplementary Figure S11B). Interestingly, these two glycine residues are also conserved in bacterial ribosomes. To test the hypothesis if rigidifying the backbone conformations of these glycine residues affect protein synthesis in yeast ribosomes, we introduced G→A substitutions to the Gly33 and Gly36. The backbone dihedral angles of Gly33 and Gly36 are in the α and Lα regions of the Ramachandran plot, respectively. Molecular modelling suggests that alanine substitutions at Gly33 and Gly36 should rigidify their backbone conformations without creating steric clashes. We also introduced G→P substitutions to these conserved glycine residues, which are expected to disrupt the local backbone conformations.

Using the yeast mutagenesis strategy described in Figure 4A, plasmids encoding variants of T7-tagged uL11 were transformed to KBeL2 strain to create the KBeL3-G33A, KBeL3-G33P, KBeL3-G36A, KBeL3-G36P strains. The balancing plasmid pRPL12B-myc in KBeL3 strains were removed by 5′FOA counterselection to create the respective KBeL4 mutant strains. Western blot analysis showed that the KBeL4 mutant strains only expressed the T7-tagged uL11 variants (Figure 5C). Yeast ribosomes were purified from KBeL4 strains (Supplementary Figure S6C), and the *in vitro* poly-phenylalanine synthesis was assayed (Figure 5D). Our results showed that G33A, G33P, G36A and G36P substitutions reduced the poly-phenylalanine synthesis activity to 27%, 9%, 27% and 32% of wild-type activity (Figure 5D).

## DISCUSSION

Ribosomal proteins uL11 is located at the base of the lateral stalk of ribosomes and forms part of the GTPase-associated center where translation factors are recruited and activated (1,2). That uL11 is involved in domain-specific action of translation factors came from the observation that replacing the bacterial stalk proteins with eukaryotic uL11 and P-complex in a bacterial ribosome change its specificity to use eukaryotic elongation factors for translation elongation (3). Structural studies of eukaryotic ribosomes show that uL11-NTD and uL10-EXT domains are involved in binding various translation factors (2,21,76–78).

Here, we have determined the NMR structure of human uL11 and showed that the linker region between the NTD and CTD of uL11 is disordered in its free form and only becomes structured when uL11 is ribosome-bound. As a result, the relative orientation of NTD and CTD of human uL11 is only fixed when it is bound to the ribosome (Figure 1). Similarly, the linker region of archaeal uL11 also has significant restructuring so that the NTD and CTD adopt different relative orientations in its free (PDB: 5COL) and the rRNA-bound form (PDB: 5DAR) (68). Since translation factors have different shapes, uL11 and uL10 must be dynamic enough to change their conformations at different states of protein synthesis to accommodate the incoming translation factors. In bacterial ribosomes, the NTD of uL11 is connected to the CTD by a flexible linker and can move relative to the CTD upon binding different translation factors (79). Structural comparison of bacterial ribosomes bound with tRNA/EF-Tu (80) and that with EF-G (81) shows that in addition to the relative domain movement, the GxxG motif of bacterial uL11 located at the C-terminus of helix-1 undergoes subtle local structural changes upon binding different translation factors (Supplementary Figure S12). In eukaryotic and archaeal ribosomes, the conserved sequence of this motif is GPLG (Figure 5A). The role of this motif in protein synthesis is supported by mutagenesis in this study. We show that rigidifying (G33A, G36A) or disrupting (G33P, G36P) the local structure of the GPLG motif in helix-1 reduced poly-phenylalanine synthesis in yeast ribosomes (Figure 5). Moreover, since the G→A substitutions are expected to rigidify the local structure without creating steric clashes, our results would suggest that the conserved glycine residues could facilitate the conformational changes of uL11 upon binding different translation factors.

**Figure 6.**
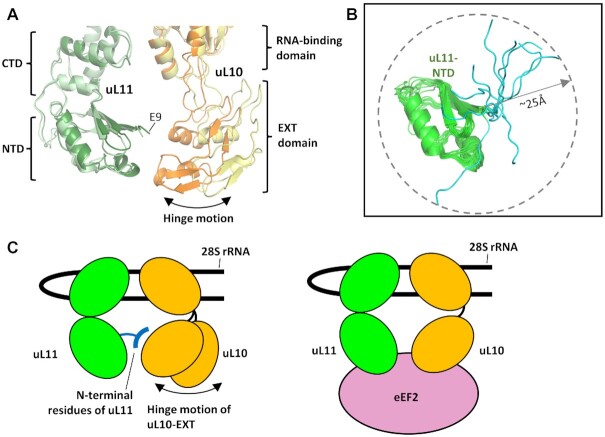
How the N-terminal residues of uL11 may play a role in protein synthesis. (**A**) The EXT domain of uL10 can undergo a hinge motion. Structures of uL11 and uL10 from the eEF2-bound 80S ribosomes (PDB: 6MTD) are superimposed with those from the eRF1/ABCE1-bound ribosomes (PDB: 5LZV). Residues 1–8 of uL11 are disordered and the location of Glu9 is indicated. uL10 and uL11 in eEF2-bound ribosomes are color-coded in darker shades of orange and green, respectively. (**B**) The ensembles of the NMR structure of NTD of uL11 (green) are superimposed. The conserved N-terminal residues (1–7, cyan) unique in eukaryotic uL11 can reach up to ∼25 Å from the NTD of uL11. (**C**) In the absence of translation factors, both the NTD of uL11 and EXT domain of uL10 are flexible. By forming transient interactions with uL10, the uL11 conserved N-terminal residues could fetch and fix uL10 into a position ready for binding the incoming eEF2 and facilitate protein synthesis. Molecular graphics were created using the program PyMOL.

In eukaryotic ribosomes, both the uL11-NTD and uL10-EXT domains are disordered when no translation factor is bound in the post-translocation state (Supplementary Figure S1D). The two ribosomal proteins become better-defined when ribosomes are bound with eEF2 (2,76) or with eRF1/ABCE1 (21) (Supplementary Figure S1). Comparison of structures of uL11 and uL10 from eEF2-bound and eRF1/ABCE1-bound ribosomes suggests that the uL10-EXT domain could undergo a rigid-body hinge motion relative to the uL11 and the RNA-binding domain of uL10 (Figure 6A).

The solution structure of human uL11 suggests that the conserved N-terminal residues are unstructured (Figure 1). ^15^N relaxation experiments further support that these residues are flexible in solution (Figure 2). In the cryo-EM structures of eukaryotic ribosomes, the first eight residues of uL11 have poor densities and were not modelled (2,21,76–78) (Supplementary Figure S1). These observations suggest that the N-terminal residues of uL11 are intrinsically disordered both in free and in ribosome-bound states.

As the N-terminal residues of uL11 (MPPKFDP) are highly conserved and unique in eukaryotes (Figure 3A), it is intriguing what could be the role of these intrinsically disordered residues in protein synthesis. We have previously established protocols to purify the human P-complex, uL10(P1/P2)_2_, and study its structural organization *in vitro* (6,7). In this study, we showed that the uL11 can interact with the P-complex *in vitro* and such interaction was lost when the conserved N-terminal MPPKFDP motif was truncated in the uL11ΔN7 variant (Figure 3B). To test if the N-terminal residues are important to protein synthesis, we prepare hybrid ribosomes by adding purified samples of human uL11 (wild-type and the ΔN7 variant) and/or P-complex to *E. coli* 50S core particles. Our results showed that poly-phenylalanine synthesis was reduced in hybrid ribosomes prepared with the uL11ΔN7 variant and P-complex (Figure 3E), suggesting the N-terminal residues of uL11 are important in translation elongation. Consistent with this suggestion, we further showed that poly-phenylalanine synthesis was also reduced in yeast ribosomes with the uL11ΔN7 variant (Figure 4D).

To understand how the N-terminal residues of uL11 may play a role in protein synthesis, we superimposed the ensemble of NMR structure of uL11 and showed that the flexible N-terminal residues could extend up to ∼25 Å from the NTD of uL11 and reach the nearby uL10 (Figure 6B). We argue that in the absence of translation factors, both the NTD of uL11 and the EXT domain of uL10 are flexible (Supplementary Figure S1D). The conserved N-terminal residues of uL11 could form transient interactions with uL10 that helps to fetch and fix uL10 into a position ready for recruiting the incoming translation factors (e.g. eEF2) and facilitate protein synthesis (Figure 6C). Truncating the N-terminal residues of uL11 could render the uL11 and uL10 too dynamic and increase the chance of non-productive collision between the incoming translation factors and the GTPase-associated center, and resulted in reduced translation elongation. How the N-terminal residues of uL11 could interact with uL10 is suggested by MD simulation. In one of the simulations, the N-terminal hydrophobic residues of uL11 are making interactions with the uL10-EXT domain (Supplementary Figure S5D). That hydrophobic residues of uL11 are important in interaction with uL10 was supported by *in vitro* pull-down assay (Supplementary Figures S2 and S3).

We would like to point out that the seven N-terminal residues (MPPKFDP) are unique in eukaryotic uL11 but not found in the archaeal uL11 (Figure 3A), while the EXT domain of uL10 is found in both eukaryotic and archaeal ribosomes. As the truncation of the N-terminal residues resulted in ∼40% poly-phenylalanine synthesis but was not lethal in yeast, it is conceivable that archaeal uL11, lacking the conserved N-terminal residues, could still function in ribosomes, but at relative lower efficiency compared with eukaryotic uL11. It is likely that the MPPKFDP motif is a later addition to the uL11 during evolution to improve the efficiency of protein synthesis in eukaryotic ribosomes.

## DATA AVAILABILITY

Atomic coordinates and NMR restraints for the refined structures have been deposited with PDB ID 7VB2 (https://www.rcsb.org/) and BMRB ID 36442 (https://bmrb.io/).

## Supplementary Material

gkac292_Supplemental_Files
