# Supplementary material for: The flexible N-terminal motif of uL11 unique to eukaryotic ribosomes interacts with P-complex and facilitates protein translation
Source: Nucleic Acids Res. 2022 May 11;50(9):5335–48. doi: 10.1093/nar/gkac292 (PMC9122527; doi:10.1093/nar/gkac292)
Supplement: gkac292_Supplemental_Files [file gkac292_supplemental_files.zip › SI.pdf]

Supplementary Information

The flexible N-terminal motif of uL11 unique to eukaryotic ribosomes interacts with P-complex and facilitates protein translation

Lei Yang [1,#], Ka-Ming Lee [1,#], Conny Wing-Heng Yu [1], Hirotatsu Imai [2], Andrew Kwok-Ho Choi [1], David K. Banfield [4], Kosuke Ito [2], Toshio Uchiumi [2,3], Kam-Bo Wong [1,*]

[1] School of Life Sciences, Centre for Protein Science and Crystallography, State Key Laboratory of Agrobiotechnology, The Chinese University of Hong Kong, Shatin, Hong Kong, China

[2] Department of Biology, Faculty of Science, Niigata University, Ikarashi 2-8050, Nishi-ku, Niigata 950-2181, Japan

[3] The Institute of Science and Technology, Niigata University, Ikarashi 2-8050, Nishi-ku, Niigata 950-2181, Japan

[4] Division of Life Science, Hong Kong University of Science and Technology, Clear Water Bay, Hong Kong, China.

# these authors contribute equally to the work

* corresponding author: email: kbwong@cuhk.edu.hk

Supplementary Table 1. Plasmids for *S. cerevisiae*

plasmid	description	source
pRS416	*CEN, URA3*	ref (32)
pRS415	*CEN, LEU2*	ref (32)
pRPL12B-myc	pRS416 carrying *RPL12B* gene with a C-terminal c-Myc tag under the control of *ADH1* promoter and terminator	This study
pRPL12B-T7	pRS415 carrying *RPL12B* gene with a C-terminal T7 tag under the control of *ADH1* promoter and terminator	This study
p4339	pCRII-TOPO::*natRMX4*	ref (63)
pRPL12B-T7-ΔN7	Deletion of the first seven residues (MPPKFDP) was made by site directed mutagenesis within uL11 coding sequence in pRPL12B-T7	This study
pRPL12B-T7-G33A	G33A substitution made by site-directed mutagenesis within the uL11 coding sequence in pRPL12B-T7	This study
pRPL12B-T7-G33P	G33P substitution made by site-directed mutagenesis within the uL11 coding sequence in pRPL12B-T7	This study
pRPL12B-T7-G36A	G36A substitution made by site-directed mutagenesis within the uL11 coding sequence in pRPL12B-T7	This study
pRPL12B-T7-G36P	G36P substitution made by site-directed mutagenesis within the uL11 coding sequence in pRPL12B-T7	This study

Supplementary Table 2. *S. cerevisiae* Strains Used in This Study

strain	description	source
Y04254	BY4741; *MATa his3Δ1 leu2Δ0 met15Δ0 ura3Δ0 rpl12B::kanMX4*	Euroscarf
KBeL1	*MATa his3Δ1 leu2Δ0 met15Δ0 ura3Δ0 rpl12B::kanMX4*, pRPL12B-myc [*CEN URA3* P_{ADH1} *RPL12B*-myc]	This study
KBeL2	*MATa his3Δ1 leu2Δ0 met15Δ0 ura3Δ0 rpl12B::kanMX4* rpl12A::*natRMX4*, pRPL12B-myc [*CEN URA3* P_{ADH1} *RPL12B*-myc]	This study
KBeL3-WT	*MATa his3Δ1 leu2Δ0 met15Δ0 ura3Δ0 rpl12B::kanMX4 rpl12A::natRMX4,* pRPL12B-myc [*CEN URA3* P_{ADH1} *RPL12B*-myc], pRPL12B-T7 [*CEN URA3* P_{ADH1} *RPL12B*-T7]	This study
KBeL4-WT	*MATa his3Δ1 leu2Δ0 met15Δ0 ura3Δ0 rpl12B::kanMX4 rpl12A::natRMX4*, pRPL12B-T7 [*CEN URA3* P_{ADH1} *RPL12B*-T7]	This study
KBeL3-ΔN7	*MATa his3Δ1 leu2Δ0 met15Δ0 ura3Δ0 rpl12B::kanMX4 rpl12A::natRMX4*, pRPL12B-myc [*CEN URA3* P_{ADH1} *RPL12B*-myc], pRPL12B-T7-ΔN7 [*CEN URA3* P_{ADH1} *RPL12B*(ΔN7)-T7]	This study
KBeL4-ΔN7	*MATa his3Δ1 leu2Δ0 met15Δ0 ura3Δ0 rpl12B::kanMX4 rpl12A::natRMX4*, pRPL12B-T7-ΔN7 [*CEN URA3* P_{ADH1} *RPL12B*(ΔN7)-T7]	This study
KBeL3-G33A	*MATa his3Δ1 leu2Δ0 met15Δ0 ura3Δ0 rpl12B::kanMX4 rpl12A::natRMX4*, pRPL12B-myc [*CEN URA3* P_{ADH1} *RPL12B*-myc], pRPL12B-T7-G33A [*CEN URA3* P_{ADH1} *RPL12B*(G33A)-T7]	This study
KBeL4-G33A	*MATa his3Δ1 leu2Δ0 met15Δ0 ura3Δ0 rpl12B::kanMX4 rpl12A::natRMX4*, pRPL12B-T7-G33A [*CEN URA3* P_{ADH1} *RPL12B*(G33A)-T7]	This study
KBeL3-G33P	*MATa his3Δ1 leu2Δ0 met15Δ0 ura3Δ0 rpl12B::kanMX4 rpl12A::natRMX4*, pRPL12B-myc [*CEN URA3* P_{ADH1} *RPL12B*-myc], pRPL12B-T7-G33P [*CEN URA3* P_{ADH1} *RPL12B*(G33P)-T7]	This study
KBeL4-G33P	*MATa his3Δ1 leu2Δ0 met15Δ0 ura3Δ0 rpl12B::kanMX4 rpl12A::natRMX4*, pRPL12B-T7-G33P [*CEN URA3* P_{ADH1} *RPL12B*(G33P)-T7]	This study
KBeL3-G36A	*MATa his3Δ1 leu2Δ0 met15Δ0 ura3Δ0 rpl12B::kanMX4 rpl12A::natRMX4*, pRPL12B-myc [*CEN URA3* P_{ADH1} *RPL12B*-myc], pRPL12B-T7-G36A [*CEN URA3* P_{ADH1} *RPL12B*(G36A)-T7]	This study
KBeL4-G36A	*MATa his3Δ1 leu2Δ0 met15Δ0 ura3Δ0 rpl12B::kanMX4 rpl12A::natRMX4*, pRPL12B-T7-G36A [*CEN URA3* P_{ADH1} *RPL12B*(G36A)-T7]	This study

KBeL3-G36P	*MATa his3Δ1 leu2Δ0 met15Δ0 ura3Δ0 rpl12B::kanMX4 rpl12A::natRMX4*, pRPL12B-myc [*CEN URA3* P_{ADH1} *RPL12B*-myc], pRPL12B-T7-G36P [*CEN URA3* P_{ADH1} *RPL12B*(G36P)-T7]	This study
KBeL4-G36P	*MATa his3Δ1 leu2Δ0 met15Δ0 ura3Δ0 rpl12B::kanMX4 rpl12A::natRMX4*, pRPL12B-T7-G36P [*CEN URA3* P_{ADH1} *RPL12B*(G36P)-T7]	This study

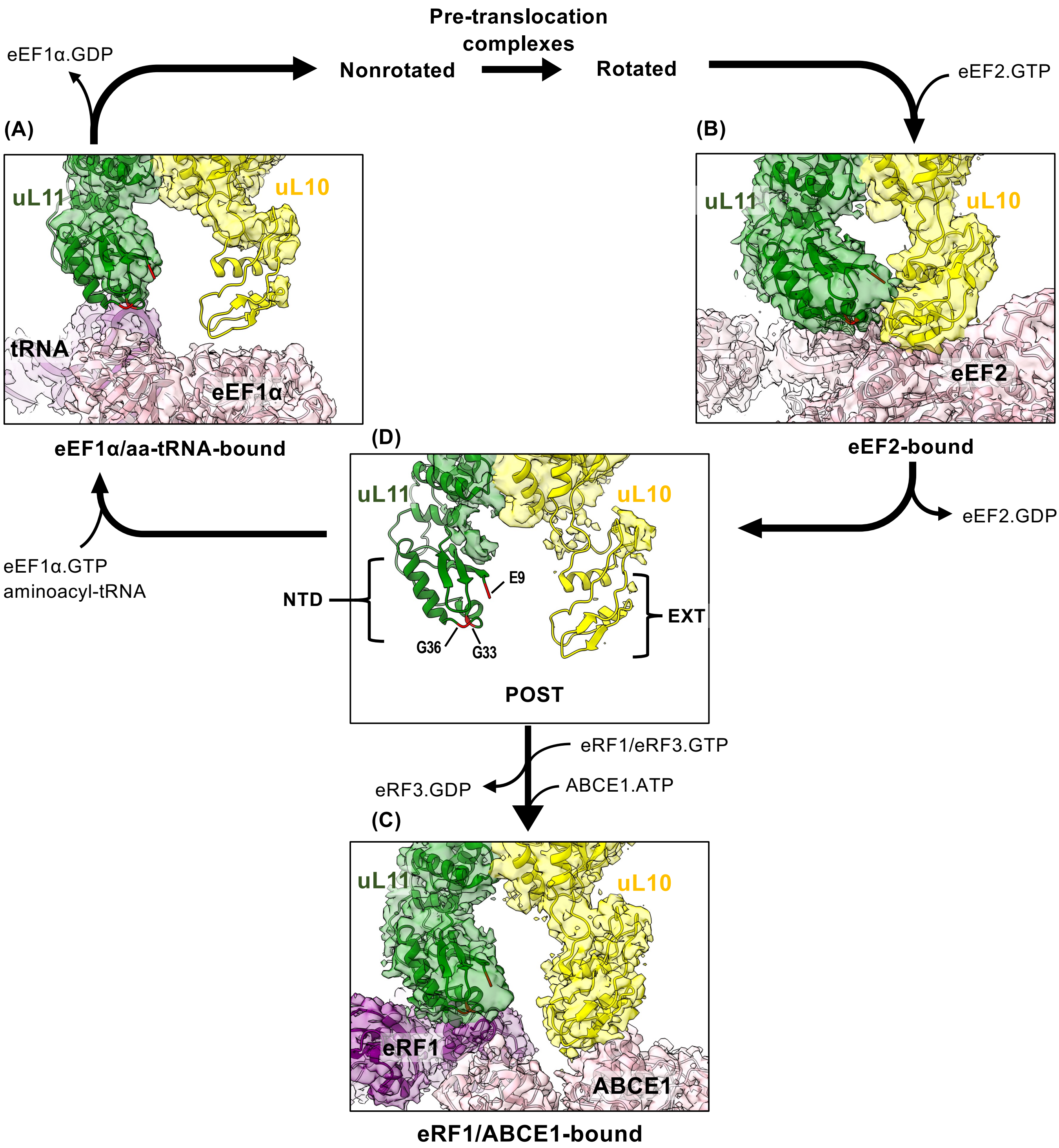

Figure S1. Structures of uL11 and uL10 in various states of eukaryotic ribosomes.
Cryo-EM structures of eukaryotic 80S ribosomes in complex with (A) eEF1α/aminoacyl-tRNA (PDB: 5LZS), (B) eEF2/SERBP1 (PDB: 6MTD), (C) eRF1/ABCE1 (PDB: 5LZV) and (D) post-translocation state (POST) (PDB: 6R5Q) are shown. Cryo-EM densities are contoured at 1σ. In all structures, residues 1-8 of uL11 are disordered and the modelled structures of uL11 start at Glu9 (color-coded red). In the POST state, both the NTD of uL11 and the EXT domain of uL10 are disordered. These two domains are better-defined in the eEF2-bound and eRF1/ABCE1-bound ribosomes. In the eEF1α/aminoacyl-tRNA bound ribosomes, the EXT domain of uL10 is disordered. The conserved Gly33 and Gly36 of uL11 are color-coded red. Molecular graphics was created using the program UCSF ChimeraX (70).

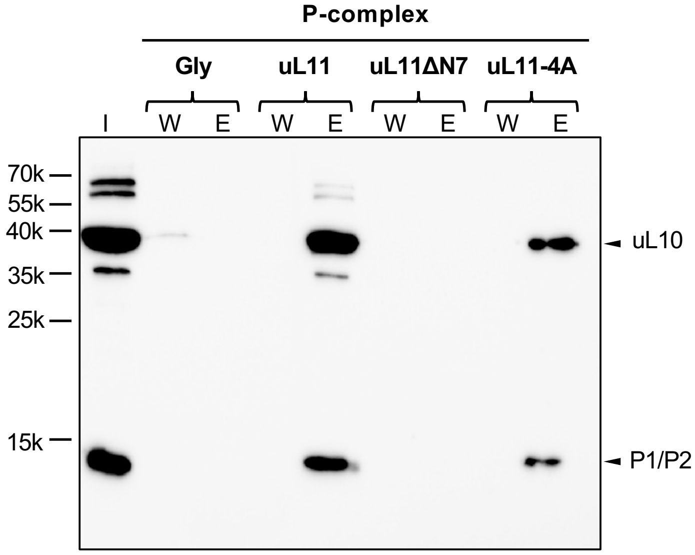

Figure S2. The N-terminal hydrophobic residues of uL11 are important for interacting with the P-complex *in vitro.*

The four N-terminal hydrophobic residues were substituted by alanine (P2A/P3A/F5A/P7A) to create the uL11-4A variant. 60 μM P-complex uL10(P1/P2)$_2$ (lane I) were loaded to NHS-resin coupled with glycine, uL11, uL11ΔN7 and uL11-4A. After extensive washing with the equilibration buffer (20mM Tris, pH 7.8) to ensure all unbound proteins were removed in the last wash fractions (lanes W), bound proteins were eluted by elution buffer (1 M NaCl, 20 mM Tris, pH 7.8) (lanes E) and analyzed by Western blot using anti-P antibody.

A

B

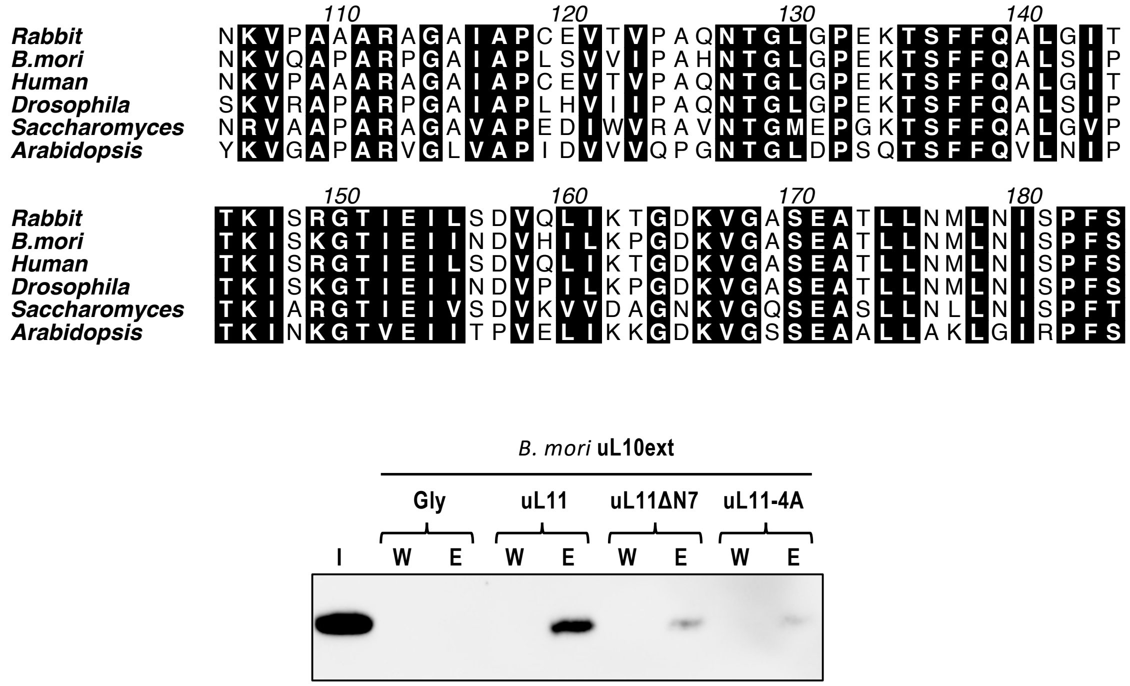

Figure S3. The N-terminal residues of uL11 interact with the EXT domain of uL10 *in vitro.*

(A) Sequence alignment uL10-EXT sequences. Residue number of the rabbit sequence is indicated at the top of the alignment. (B) 60 μM *B. mori* uL10-EXT (lane I) were loaded to NHS-resins coupled with glycine, uL11, uL11ΔN7 and uL11-4A. After extensive washing with the equilibration buffer (20mM Tris, pH 7.8) to ensure all unbound proteins were removed in the last wash fractions (lanes W), bound proteins were eluted by high salt elution buffer (1 M NaCl, 20 mM Tris, pH 7.8) (lanes E) and analysed by Western blot using uL10-EXT antibody

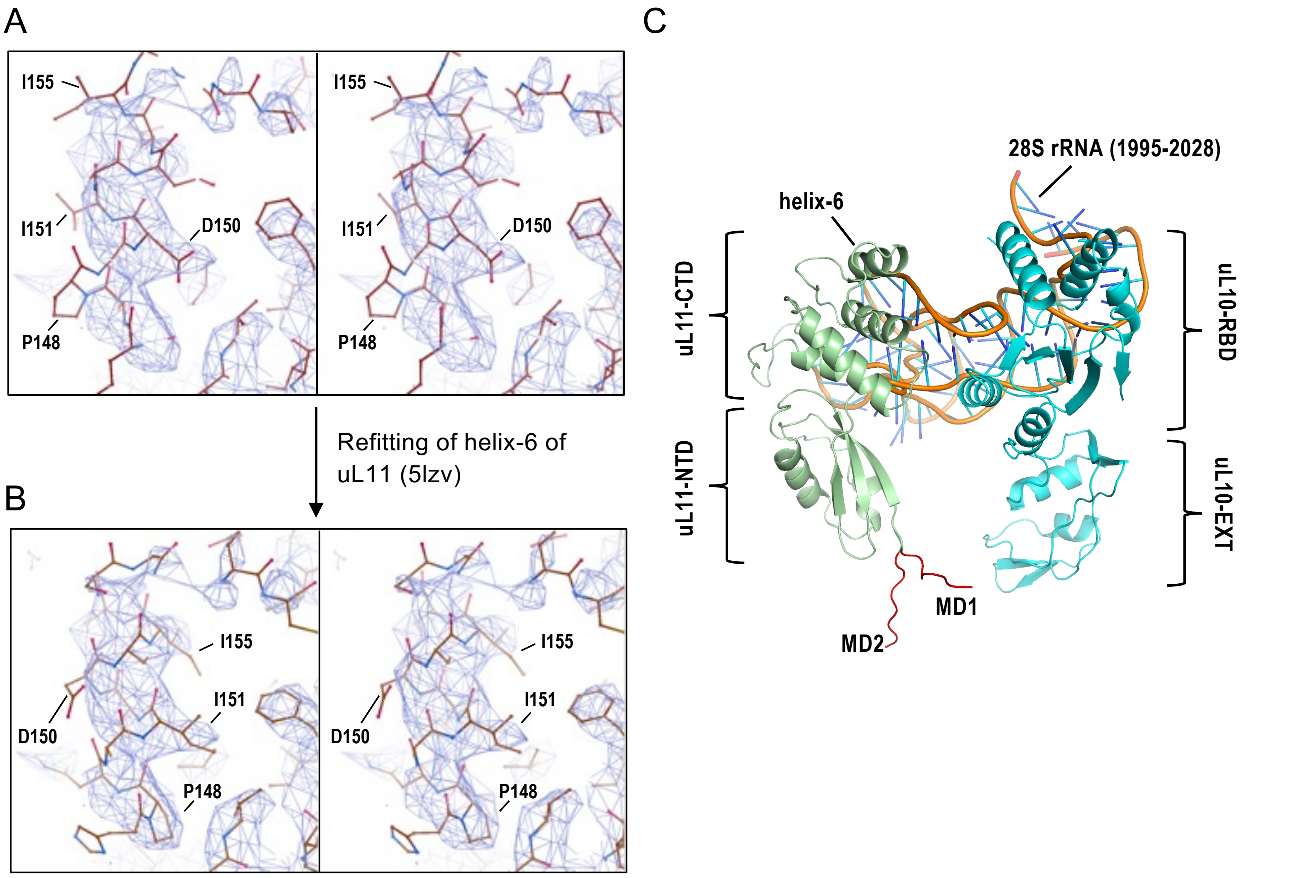

Figure S4. Preparation of the starting structures for molecular dynamics simulation. (A) In the deposited structure of ribosomes in complex with eRF1/ABCE1 (PDB: 5LZV), Asp150 of helix-6 of uL11 was located in a buried position. (B) Based on the crystal structure of uL11 of *M. jannaschii* (PDB:5DAR), helix-6 of uL11 was refitted to the cryo-EM densities of 5LZV. After refitting, hydrophobic residues Pro148, Ile151 and Ile155 are now facing the hydrophobic core of uL11, and Asp150 is on the surface of uL11. (C) The starting structures of MD simulations contain coordinates of the uL10 (cyan) and 28 rRNA (1995-2028) from 5LZV and refitted coordinates of uL11 (green) from (B). The initial structures of the N-terminal residues 2-8 of uL11 (red) were derived from the NMR structure of uL11. Two conformers from the NMR ensembles were selected to create the starting structures for MD simulations. The N-terminal methionine of uL11, which should be removed in eukaryotic ribosomes, was not included in the MD simulation.

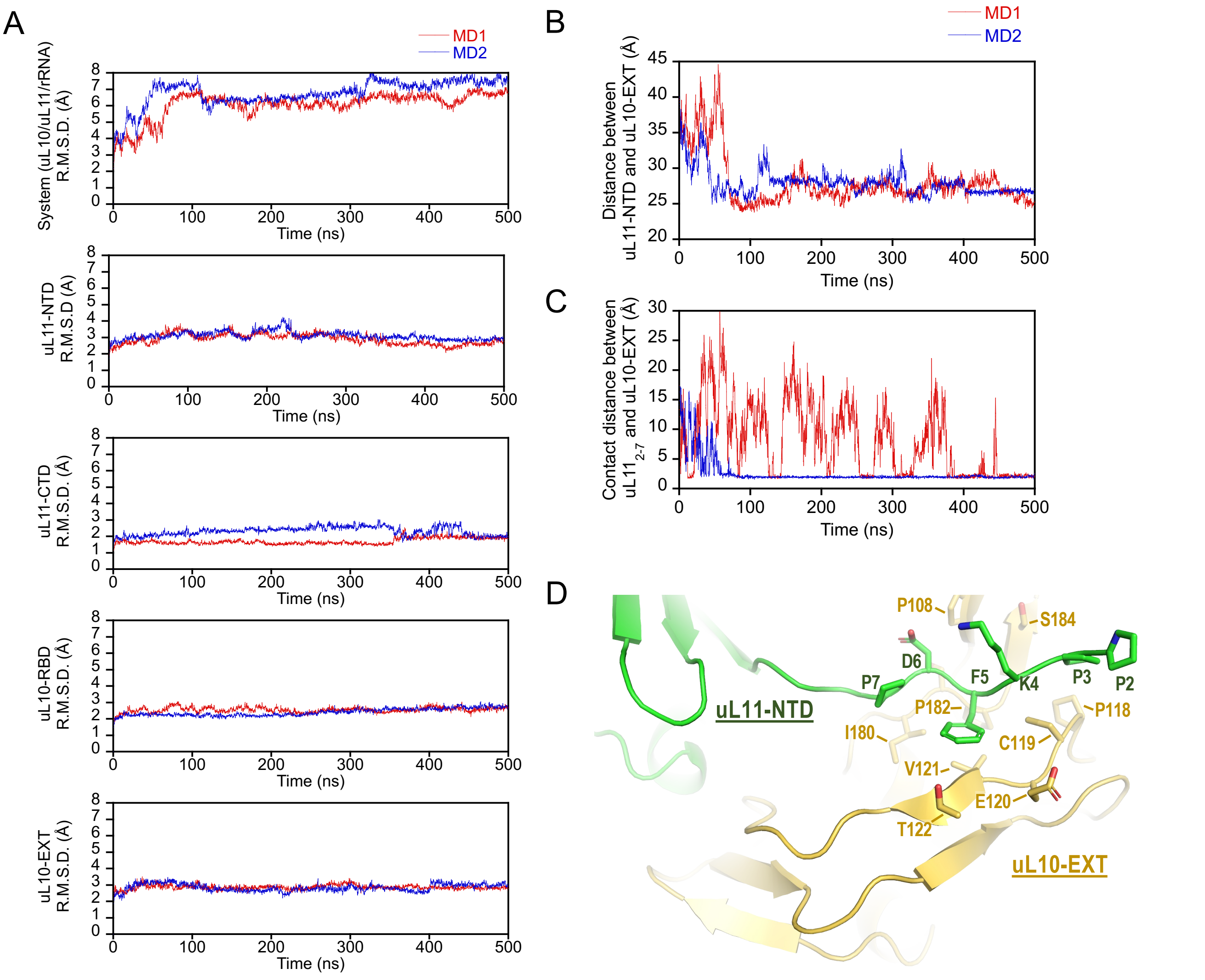

Figure S5. MD simulations may provide insights into how uL11 interacts with uL10.
(A) Root mean square deviation (R.M.S.D.) from the starting structure of heavy atoms in the system, uL11-NTD (8-75), uL11-CTD (108-158), uL10-RBD (RNA-binding domain; 5-107,185-200) and uL10-EXT (108-184). Values of MD1 and MD2 simulations were in red and blue, respectively. (B) Distance between centres of geometry of uL11-NTD and uL10-EXT as a function of simulation time. (C) Shortest distance between N-terminal residues 2-7 of uL11 and uL10-EXT as a function of simulation time. In MD1 (red), interaction between N-terminal residues of uL11 and uL10-EXT is transient. In MD2 (blue), N-terminal residues of uL11 form lingering interactions with uL10-EXT after ~100 ns. (D) Representative snapshot of how uL11 interacts with uL10-EXT at 200 ns of MD2. Residues of uL10-EXT (yellow) within 4 Å of N-terminal residues of uL11 (green) are shown.

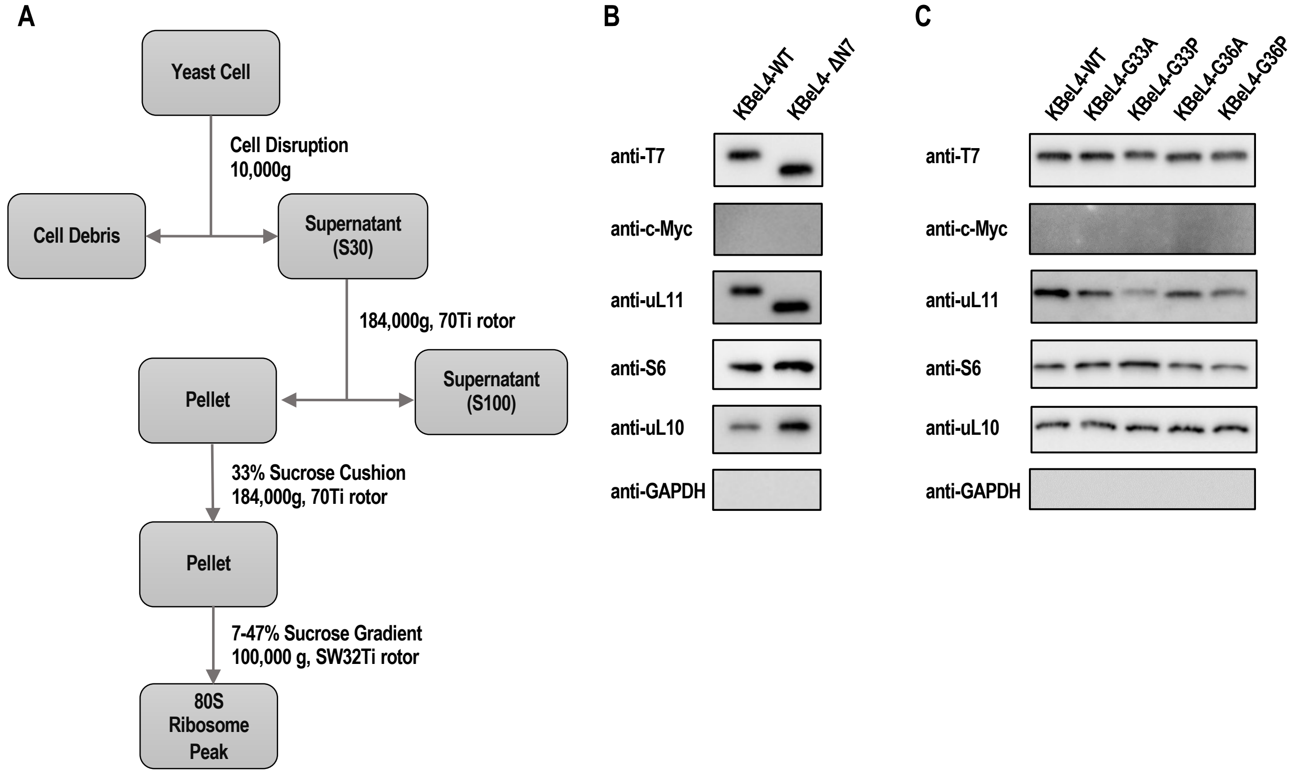

Figure S6. Purification of yeast ribosomes.

(A) Schematic diagram of the 80S ribosome purification procedures. (B) and (C) 80S yeast ribosomes were purified from yeast strains KBeL4-WT, KBeL4-ΔN7, KBeL4-G33A, KBeL4-G33P, KBeL4-G36A and KBeL4-G36P and analyzed by Western blot using T7, c-Myc, uL11, S6, uL10 and GAPDH antibodies. S6 and uL10 were selected as markers for the ribosomal small and large subunits, respectively. The purified ribosomes contained only the T7-tagged uL11 or its variants, but not the c-Myc-tagged wild-type uL11. Absence of GAPDH suggested that the cytoplasm fraction had been removed through ribosome purification processes.

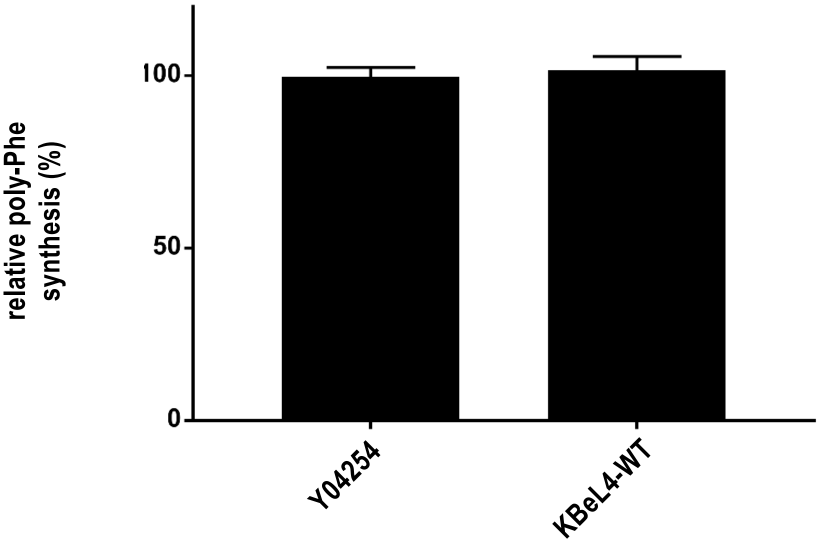

Figure S7. The T7-tag on uL11 has no effect on the protein synthesis.
Ribosomes were purified from yeast strains Y04254 (expressing the endogenous uL11) and KBeL4-WT (expressing T7-tagged uL11) and were assayed for their *in vitro* polyphenylalanine synthesis. Our results showed that there were no significant difference in poly-phenylalanine synthesis between the two yeast ribosomes.

A

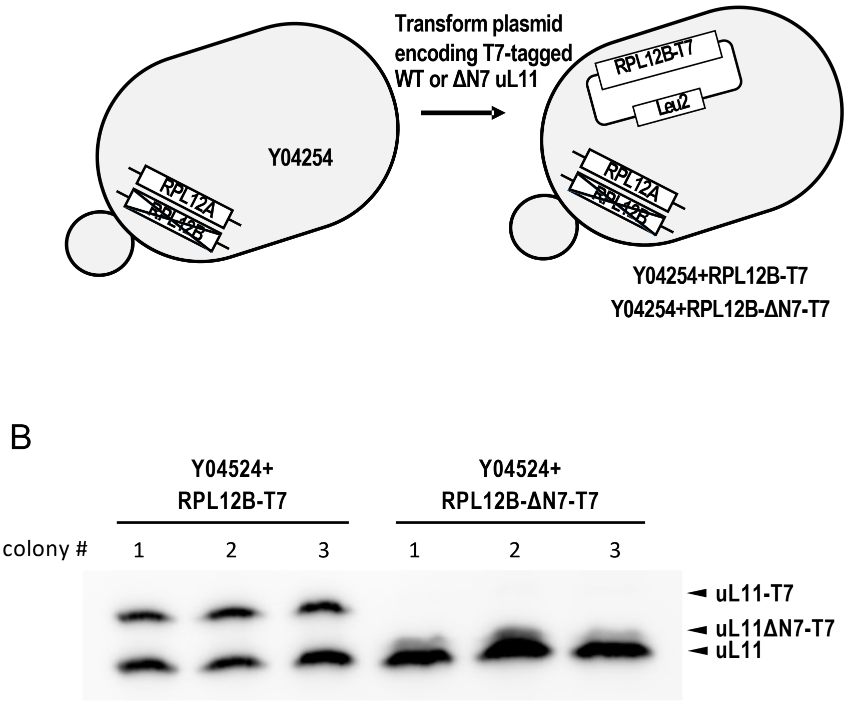

Figure S8. Expression of uL11ΔN7 was lower than wild-type uL11 in the parental Y04254 strain. (A) Plasmids encoding T7-tagged wild-type uL11 or uL11ΔN7 were transformed to the parental Y04254 strain to create Y04524+RPL12B-T7 and Y04524+RPL12B-ΔN7-T7 strains. (B) After transformation, cell lysates were analysed by Western blot using uL11 antibodies. Three colonies from each of the transformants were analysed. The results show that the expression of uL11ΔN7-T7 was consistently lower than that of the wild-type uL11 in the parental Y04254 strain.

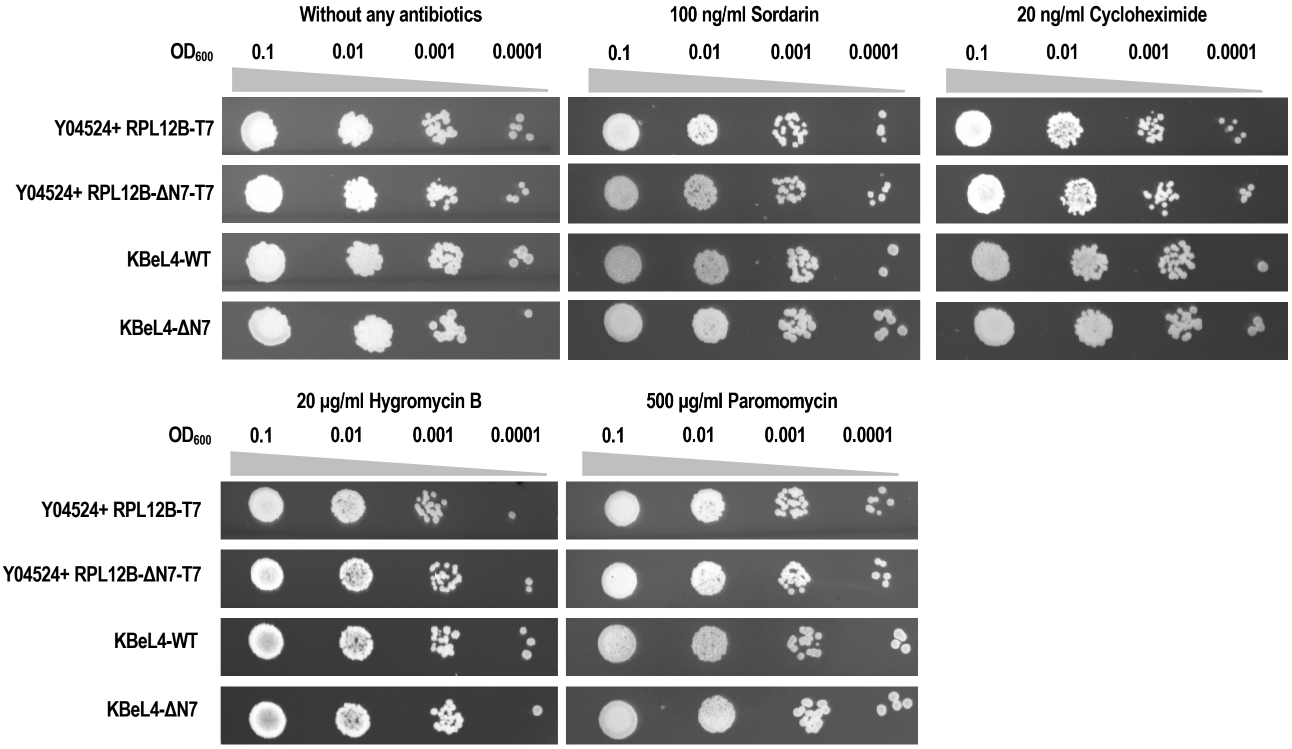

Figure S9. Antibiotics sensitivity of yeast mutant strains. 3 μl yeast culture of various concentrations (OD_{600}=0.1, 0.01, 0.001, 0.0001) was spotted onto agar plates supplemented with 4 μg/ml sordarin, 20 ng/ml cycloheximide, 20 μg/ml hygromycin B and 500 μg/ml paromomycin. Yeast growth on agar plates without any antibiotics was used as a control. The results suggest that expression of ΔN7 mutant of uL11 did not affect the antibiotics sensitivity of the yeasts.

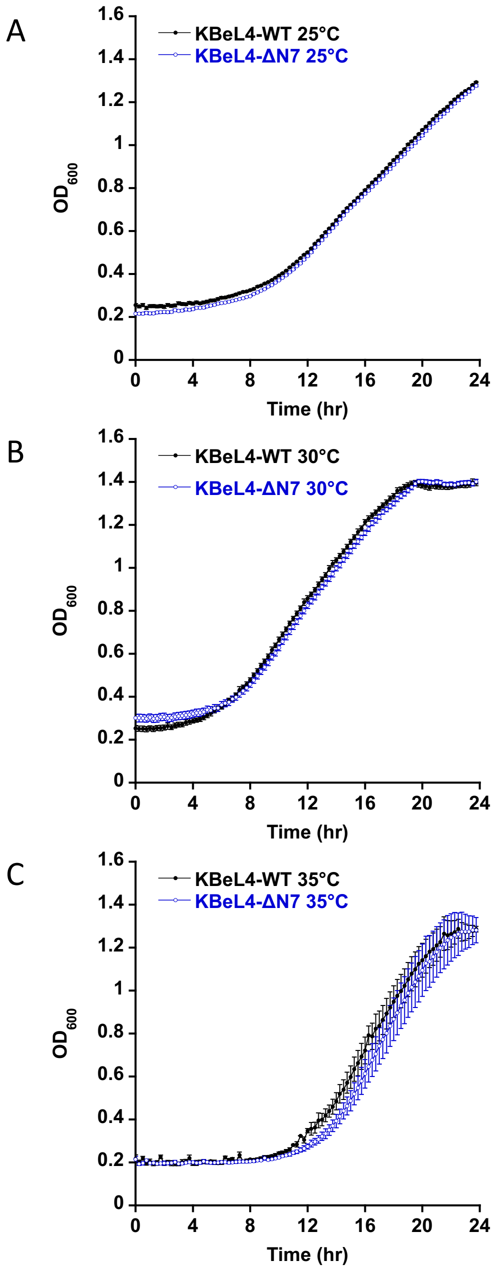

Figure S10. Growth rates of KBeL4-WT and KBeL4-ΔN7 were similar at 25˚C, 30˚C and 35˚C. The growth of KBeL4-WT and KBeL4-ΔN7 yeast strains at 25˚C, 30˚C and 35˚C was followed by OD_{600} at 25˚C, 30˚C and 35˚C. The error bars represent S.E.M. for at least three measurements. The growth rates were obtained by fitting the data in the exponential growth phase to the simple exponential growth equation using the program PRISM (GraphPad Software, LLC). The growth rates for KBeL4-WT and KBeL4-ΔN7 strains were 0.1082 ± 0.0005 and 0.1136 ± 0.0005 h^{-1} at 25˚C, 0.1492 ± 0.002 and 0.1457 ± 0.003 h^{-1} at 30˚C, and 0.191 ± 0.007 and 0.1894 ± 0.009 h^{-1} at 35˚C, respectively.

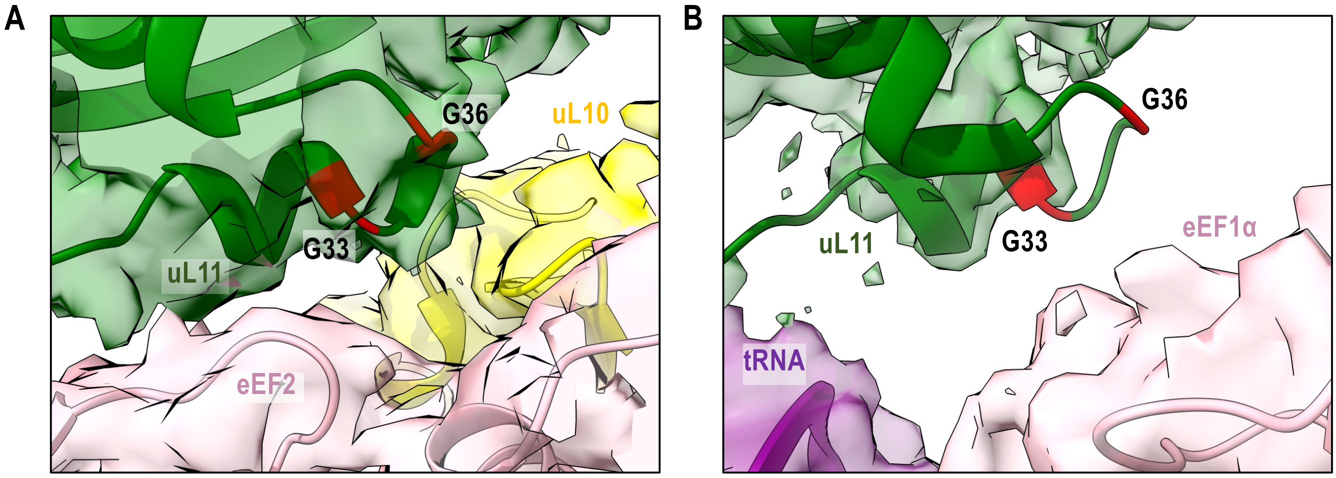

Figure S11. The $_{33}$GPLG$_{36}$ motif of uL11 is better defined in the eEF2-bound ribosomes than in the eEF1α-bound ribosomes. Cryo-EM structure of eukaryotic 80S ribosomes in complex with (A) eEF2 (pink) and SERBP1 (PDB: 6MTD), is compared to that (B) with eEF1α (pink), aminoacyl-tRNA (purple), and didemnin B (PDB: 5LZS). The conserved Gly33 and Gly36 of uL11 are color-coded red. It is noted that the density for the $_{33}$GPLG$_{36}$ motif is not defined in the eEF1α-bound state. Cryo-EM densities are contoured at 1σ. Molecular graphics was created using the program UCSF ChimeraX (70).

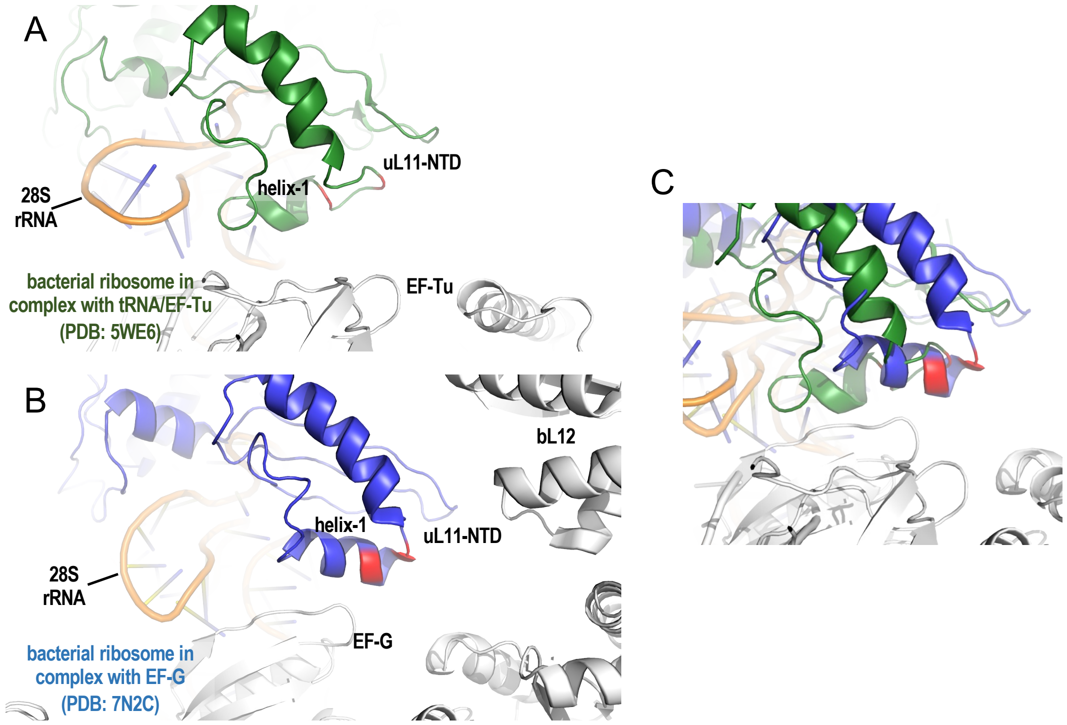

Figure S12. Structural comparison of uL11 in bacterial ribosomes. Cryo-EM structure of bacterial ribosomes bound with (A) tRNA/EF-Tu (PDB:5WE6) is compared to that (B) with EF-G (PDB: 7N2C). The two structures are superimposed in panel C. Bacterial uL11 also contains a conserved GxxG motif at the C-terminus of helix-1. The conserved glycine residues of the motif are colored in red.
